# Gene expression profiles at different stages for formation of pearl sac and pearl in the pearl oyster *Pinctada fucata*

**DOI:** 10.1186/s12864-019-5579-3

**Published:** 2019-03-25

**Authors:** Mariom, Saori Take, Yoji Igarashi, Kazutoshi Yoshitake, Shuichi Asakawa, Kaoru Maeyama, Kiyohito Nagai, Shugo Watabe, Shigeharu Kinoshita

**Affiliations:** 1 Graduate School of Agricultural and Life Sciences, The University of Tokyo, Bunkyo, Tokyo, 113-8657 Japan; 2 Department of Fisheries Biology and Genetics, Faculty of Fisheries, Bangladesh Agricultural University, Mymensingh, 2202 Bangladesh; 3 Mikimoto Pharmaceutical CO., LTD, Kurose 1425, Ise, Mie 516-8581 Japan; 4 Pearl Research Laboratory, K. MIKIMOTO & CO., LTD, Osaki Hazako 923, Hamajima, Shima, Mie 517-0403 Japan; 5 School of Marine Biosciences, Kitasato University, Minami, Sagamihara, Kanagawa 252-0313 Japan

**Keywords:** Gene expression, Different stages, Pearl sac, Pearl, *Pinctada fucata*

## Abstract

**Background:**

The most critical step in the pearl formation during aquaculture is issued to the proliferation and differentiation of outer epithelial cells of mantle graft into pearl sac. This pearl sac secretes various matrix proteins to produce pearls by a complex physiological process which has not been well-understood yet. Here, we aimed to unravel the genes involved in the development of pearl sac and pearl, and the sequential expression patterns of different shell matrix proteins secreted from the pearl sac during pearl formation by pearl oyster *Pinctada fucata* using high-throughput transcriptome profiling.

**Results:**

Principal component analysis (PCA) showed clearly different gene expression profiles between earlier (before 1 week) and later stages (1 week to 3 months) of grafting. Immune-related genes were highly expressed between 0 h – 24 h (donor dependent) and 48 h – 1 w (host dependent), and in the course of wound healing process pearl sac was developed by two weeks of graft transplantation. Moreover, for the first time, we identified some stem cell marker genes including *ABCG2, SOX2, MEF2A, HES1, MET, NRP1, ESR1, STAT6, PAX2, FZD1* and *PROM1* that were expressed differentially during the formation of pearl sac. The expression profiling of 192 biomineralization-related genes demonstrated that most of the shell matrix proteins (SMPs) involved in prismatic layer formation were first up-regulated and then gradually down-regulated indicating their involvement in the development of pearl sac and the onset of pearl mineralization. Most of the nacreous layer forming SMPs were up-regulated at 2 weeks after the maturation of pearl sac. Nacrein, MSI7 and shematrin involved in both layer formation were highly expressed during 0 h – 24 h, down-regulated up to 1 week and then up-regulated again after accomplishment of pearl sac formation.

**Conclusions:**

Using an RNA-seq approach we unraveled the expression pattern of the key genes involved in the development of pearl sac and pearl as a result of host immune response after grafting. These findings provide valuable information in understanding the molecular mechanism of pearl formation and immune response in *P. fucata*.

**Electronic supplementary material:**

The online version of this article (10.1186/s12864-019-5579-3) contains supplementary material, which is available to authorized users.

## Background

The bivalve mollusk, *Pinctada fucata*, is well known throughout the world for its ability of producing high quality pearl and accounts for more than 90% of seawater pearl production [1]. Artificial pearl production using this species was first industrialized in late 1890s in Japan [2].

In pearl farming, a small piece of mantle tissue from a donor oyster is implanted into the gonad of a host oyster along with an inorganic bead (termed as ‘nucleus’) for nucleated-pearl production [3, 4]. The outer surface of this mantle graft is covered by a monolayer of ciliated columnar epithelial cells with basal nuclei that undergoes proliferation and differentiation into a layer of secretory epithelium encircling the nucleus called ‘pearl sac’ [5, 6]. The outer epithelium should contain proliferative stem cells that differentiate into pearl sac afterwards, but the features of those cells are unclear. After successful implantation, the growth and development of pearl sac depends on the interactions between donor graft cells and those of host gonad tissues. The graft tissue firmly clings to the gonad tissue with the proliferation of the epithelial cells and forms a pearl sac in course of time [7]. The cells of the pearl sac obtain nourishment from the surrounding haemolymph [8]. Usually, it takes about 1 to 4 weeks to complete the development of pearl sac depending on several conditions like water temperature [9], season [10], sex of host oyster [11], species [7, 12, 13] and so on. The epidermal cells (secretory epithelium) of the fully grown pearl sac gradually secrete and deposit various matrix proteins surrounding the nucleus that eventually results in the formation of a lustrous pearl [3, 6]. The mineralization process that occurs during the formation of cultured pearl is very similar to that of inner shell biomineralization regulated by the mantle [3, 4]. Therefore, it is very reasonable to claim that pearl sac formation is the most important step of pearl culture that ultimately determines the success of culture.

The unique ability of producing pearl has made the pearl oyster one of the best-studied species in relation to biomineralization. The major biomineralization product in nature is the mollusk shell. The pearl oyster shell consists of two distinct layers: inner nacreous layer made of aragonite and outer prismatic layer made of calcite [14]. Many studies have been focused on oyster shell formation and revealed that the formation of prismatic and nacreous layer is regulated by the proteins secreted from mantle [14–16]. To date, a vast number of shell matrix proteins have been identified that play a vital role in the molecular mechanism underlying the formation of shell and pearl [16–18]. Some of these genes are involved in the formation of prismatic layer [19–22], some in nacreous layer [22–26], some in both layers [27–29], and the others control and modulate the secretion and expression of these shell or pearl forming genes [18, 30, 31]. Though the mechanism of pearl formation in *Pinctada* has been studied extensively, the complex physiological process by which pearl sac and pearl is developed is not well-understood yet.

Moreover, the surgical implantation practiced in pearl grafting can induce the immune reaction in host oyster to some extent in response to receiving a transplant and the oyster survival [32, 33]. Therefore, it is very important to explore the key genes involved in the immunological changes that occur upon graft transplantation. A transcriptome study in *P. martensii* detected some immune-related genes including *HSP90*, toll-like receptors (TLRs) and lysozyme from the pearl sac after 180 days of implantation [34]. Very recently, some studies examined the immune reaction of the pearl oyster hemocyte upon allografting [33, 35] and xenografting [36] by transcriptome analysis. Some studies also explained that the process of the pearl-sac formation during pearl culture is identical to the wound healing process that occurs after a mantle injury [37, 38]. However, the immunological reaction that appears in the donor mantle graft and in the host oyster during the subsequent stages of pearl sac formation is still unclear. Accordingly, increased understanding of the host immune response upon accepting a transplant is required to further improve the effectiveness of pearl culture technique.

With the development of versatile and cost-effective next generation sequencing technology, RNA sequencing has been extensively used in the genomic research of various organisms [39, 40]. It allows a broad genome coverage with unbiased quantification of transcript expression in order to identify important genes or pathways involved in various biological processes with their expression profiling [41, 42]. In the present study, we therefore aimed to identify the genes playing a critical role in the formation of pearl sac and pearl using high-throughput transcriptome profiling. Moreover, we identified some stem cell marker genes differentially expressed during pearl sac development. Simultaneously, we screened out the key genes involved in the immunological changes that occur during pearl sac formation. Our second goal was to improve the overall understanding of the expression profiles of 192 pearl forming genes secreted from the pearl sac epithelium during the development of pearl. Then, we focused on the detailed expression pattern of the well-known shell matrix proteins (SMPs) during three months grafting experiment. Additionally, we examined the pearl layers that deposited on the nucleus to verify the results obtained from the gene expression studies.

## Results

### Transcriptome sequence assembly

The results of statistical analysis of sequencing data are summarized in Table 1 and Additional file 1: Table S1. After filtering, the total number of clean reads was 925.35 million. The quality assessment of the sequencing data showed that the distribution of quality Q20 was more than 95% in each sample and the GC content was 39.04–50.37%. Again, 65.52% of the clean reads were successfully quantified with Kallisto [43] to obtain transcript counts and abundances (Table 1).

**Table 1:** Statistical analysis of transcriptome sequencing data

Parameters	Counts
Total clean reads	925,349,740
Total clean bases	92,534,974,000
Q20	> 95.13%
GC count	39.04–50.37%
Quantified reads (× 10^6^)	813.77
Quantification ratio	65.52%

### Clustering of samples by whole gene expression patterns

Sample distances were calculated using R and visualized in a heatmap to know the differences in overall gene expression pattern during different stages of pearl formation. Cluster analysis revealed the dissimilarities in gene expression at various stages of pearl grafting as the samples were divided into four distinct groups: cell, before – 0 h, 24 h – 48 h and 1 w – 3 m (Additional file 1: Figure S1). The figure illustrated that expression profile in cell was apart from any other groups. The differences in expression in ‘cell’ might be derived from two possible reasons. The first one is as ‘cells’ consists of only outer epithelial cells but ‘mantle pallium’ contains outer epithelial cells, inner epithelial cells, connective tissues and so on. Another is the differences in preparation technique. ‘Mantle pallium’ is just cut from the mantle whereas, ‘cell’ is prepared from mantle through a complicated process described in the method. These preparation steps might affect the gene expression in ‘cell’. Again, upto 48 h samples, the clusters were ‘stage dependent’ as the samples were separated in three different stages (cell, before – 0 h and 24 h – 48 h) (Additional file 1: Figure S1). At each stage, the clusters were also ‘donor dependent’ because the samples/grafts obtained from the same donor were grouped together. For example, in 24 h – 48 h cluster, the host oysters 24h_A1, 24h_A2, 48h_A1 and 48h_A2 received graft from the ‘donor A’ were grouped together (Additional file 1: Figure S1). On the other hand, the expression in 1 w – 3 m samples is ‘host dependent’ as the cluster for 1 w – 3 m samples is neither stage dependent nor donor dependent (Additional file 1: Figure S1). This is because after transplantation, the grafts and the later pearl sacs were contaminated with host gonad tissues especially from 1 week to 3 months samples.

**Fig. 1:**
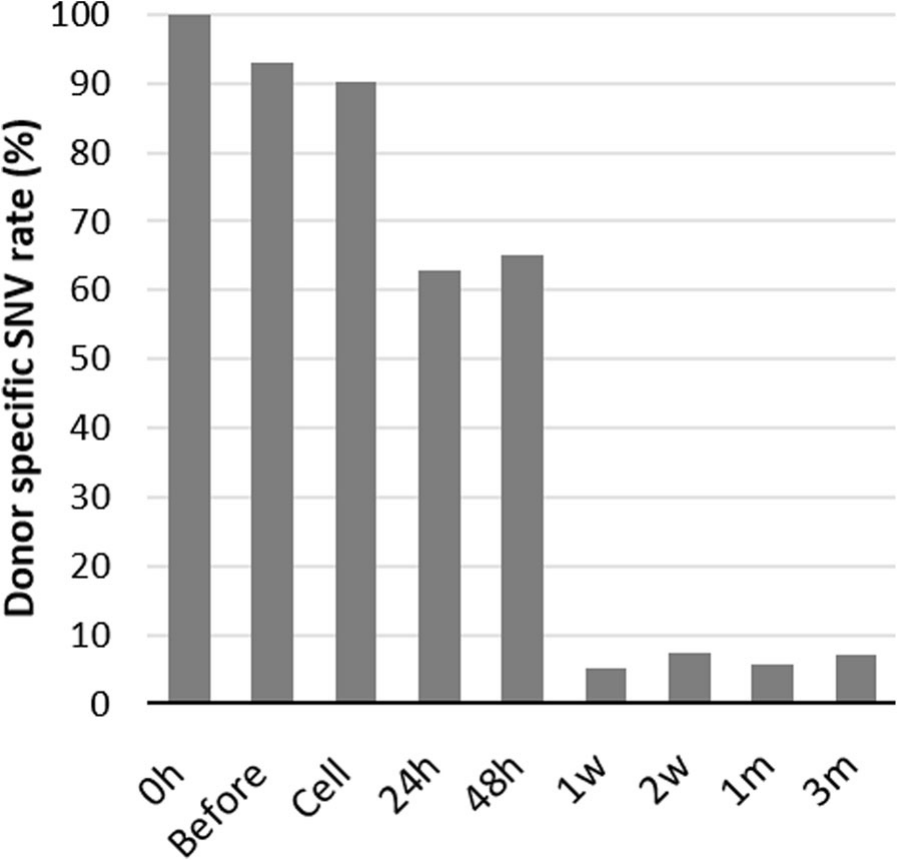
Donor specific SNV rate (%) in different samples. The X- and Y-axis illustrate samples at different time points and percentage of SNV, respectively

In order to know the rate of contamination of host cells, we therefore detected single nucleotide variants (SNVs) between donor and host transcripts. In case of xenografting from two closely related species where inter-specific sequence differences in homologous biomineralization genes are present, it is possible to discern whether the donor or host cells are transcriptionally active for the relevant gene [44]. But due to the lack of data on intra-specific polymorphisms in biomineralization genes, it is impractical to separate the gene transcripts derived from individual oysters used as donors or hosts in allografting. As we performed allografting, hence we only calculated the percentage of donor specific SNVs in each sample (Fig. 1) in order to get the actual expression of donor specific transcripts for the studied biomineralization-related genes. But in the calculation, the rate of donor specific SNVs were underestimated since not only ‘0 h’ samples but also ‘before’ and ‘cell’ samples were without any contamination with host tissues i.e. donor specific SNV rate should be 100% (Fig. 1). Hence, the real donor specific SNVs in all the samples except ‘0 h’ were little more than that showed in Fig. 1. Additionally, Fig. 1 likewise Additional file 1: Figure S1 described that 0 h – 48 h samples contained transcripts mainly from donor whereas most of the transcripts in 1 w – 3 m samples were from host.

### Functional annotation and classification of the DEGs between different time groups

**Fig. 2:**
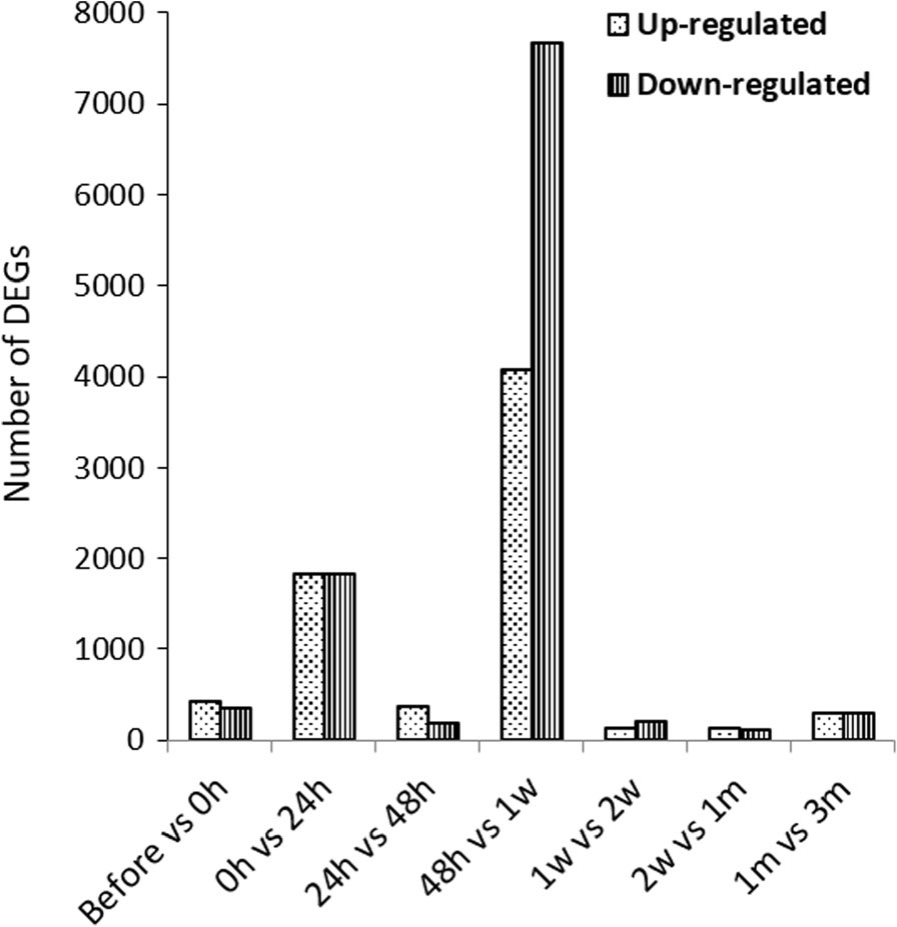
The numbers of up-regulated and down-regulated DEGs at different time points of three months grafting experiment. Before and 0 h means pre-transgraft. The others, 24 h – 3 m, mean post-transgraft. “h” for hour, “w” for week and “m” for month

To discern the successive changes that occurs during pearl formation upon grafting, we considered seven consecutive time combinations (before – 0 h, 0 h – 24 h, 24 h – 48 h, 48 h − 1 w, 1 w – 2 w, 2 w – 1 m and 1 m – 3 m) during three months grafting experiment. The total up- and down-regulated differentially expressed genes (DEGs) were detected at seven mentioned time combinations (Fig. 2). The highest number of total DEGs (11,744) was detected at 48 h – 1 w time point of which 4076 were up-regulated and 7668 were down-regulated. All the DEGs at mentioned seven time points were then used for subsequent gene ontology (GO) enrichment analysis. In GO enrichment analysis, three functional categories were determined: biological process, cellular component and molecular function (Additional file 2: Table S2a,b).

### Differentially expressed genes in immune-related pathways

**Fig. 3:**
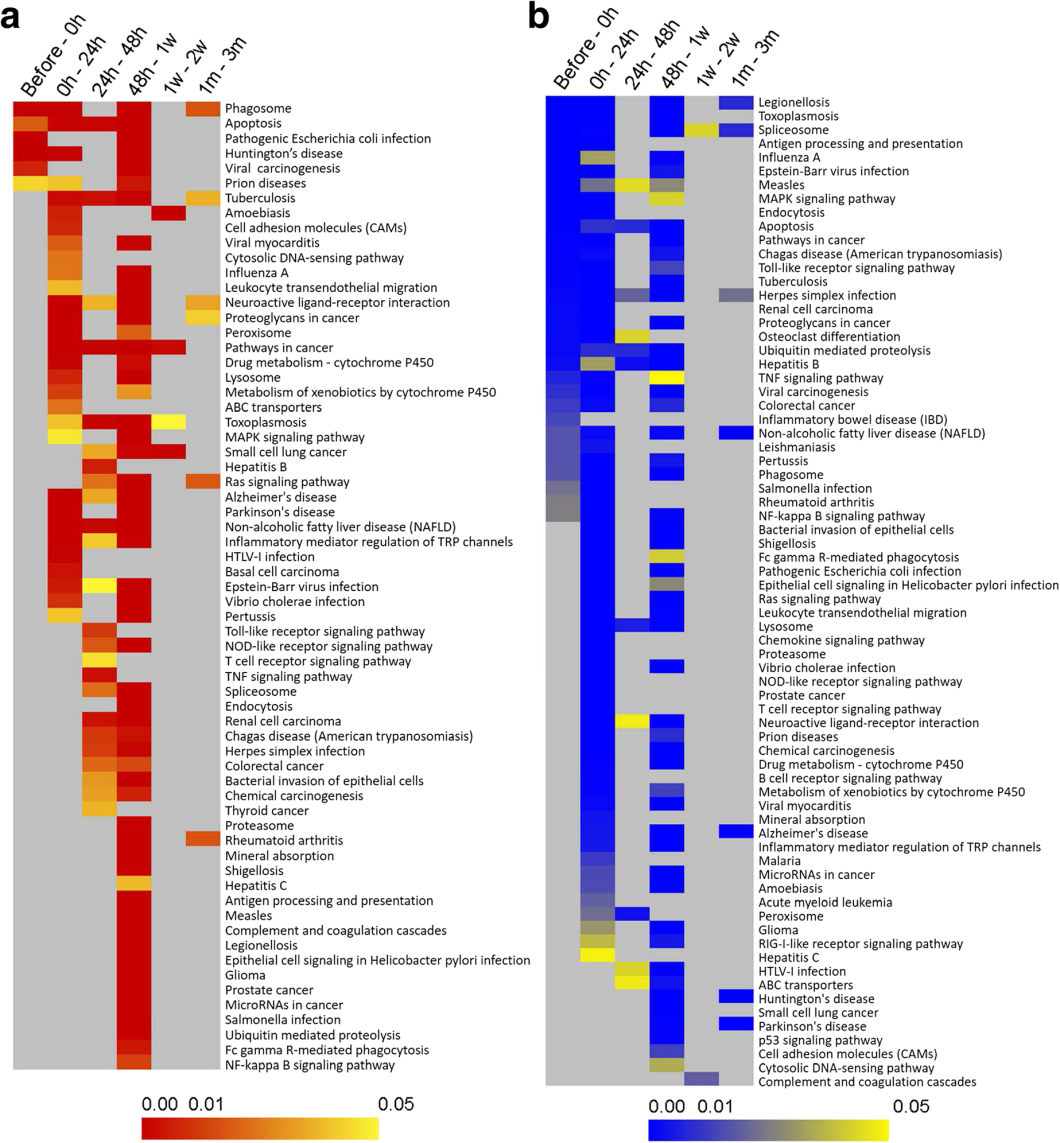
Heat maps of KEGG pathway enrichment analysis for immune related DEGs. **a** Up-regulated DEGs. **b** Down-regulated DEGs. Up- or down-regulated DEGs at each time point were submitted to KEGG pathway analysis using Kobas 3.0 web-based software. Columns and rows in the heat maps indicate treatments and enriched pathway terms, respectively. Sample names are displayed above the heat maps. Color scales indicate *P* values of enrichment tests and gray cells represent an empty value or a value > 0.05

The immune response that occurs upon grafting process during cultured pearl production plays a vital role in response to oyster survival and regeneration [33]. To gain insights into the potential functioning of immune system, we monitored the expression of the key genes in different immune-related pathways throughout the grafting period. According to the results of GO enrichment analysis, most of the immune-related genes were enriched at 0 h – 24 h and 48 h – 1 w time points. At 0 h – 24 h time point, 128 and 188 immune related terms were up- and down-regulated, respectively, whereas at 48 h – 1 w time point, 67 and 216 terms were up- and down-regulated, respectively (Additional file 3: Table S3). Further, we mapped all the DEGs in the Kyoto encyclopedia of genes and genomes (KEGG) database to search for the genes involved in significant immune-related pathways. Figure 3 explained that immune related pathways were significantly (*P* < 0.05) enriched at 0 h – 24 h and 48 h – 1 w time points which is consistent with the results of GO enrichment analysis (Additional file 3: Table S3). During graft preparation (before – 0 h) most of the immune pathways were down-regulated due to the suppression of immune genes in early donor cells i.e. before samples (Fig. 3b). After transplantation, surrounding host hemocytes encapsulate the graft and nucleus making the graft contaminated with host immune cells. Thus 0 h – 24 h comparison elucidating that host immune cells became active at the site of grafting at 24 h (Fig. 3). Most of the immune genes were enriched at 48 h – 1 w interpreting the distinction of immune functions between donor (48 h) and host (1 w) cells. From the observation of 48 h – 1 w enrichment, it is complicated to conclude whether donor or host immune cells were more functional at this stage. The up and down-regulated DEGs involved in 21 crucial immune pathways during the process of pearl sac formation were screened out by KEGG pathway analysis and listed in Additional file 4: Table S4.

### Differentially expressed genes related to epithelial cell proliferation and differentiation

**Fig. 4:**
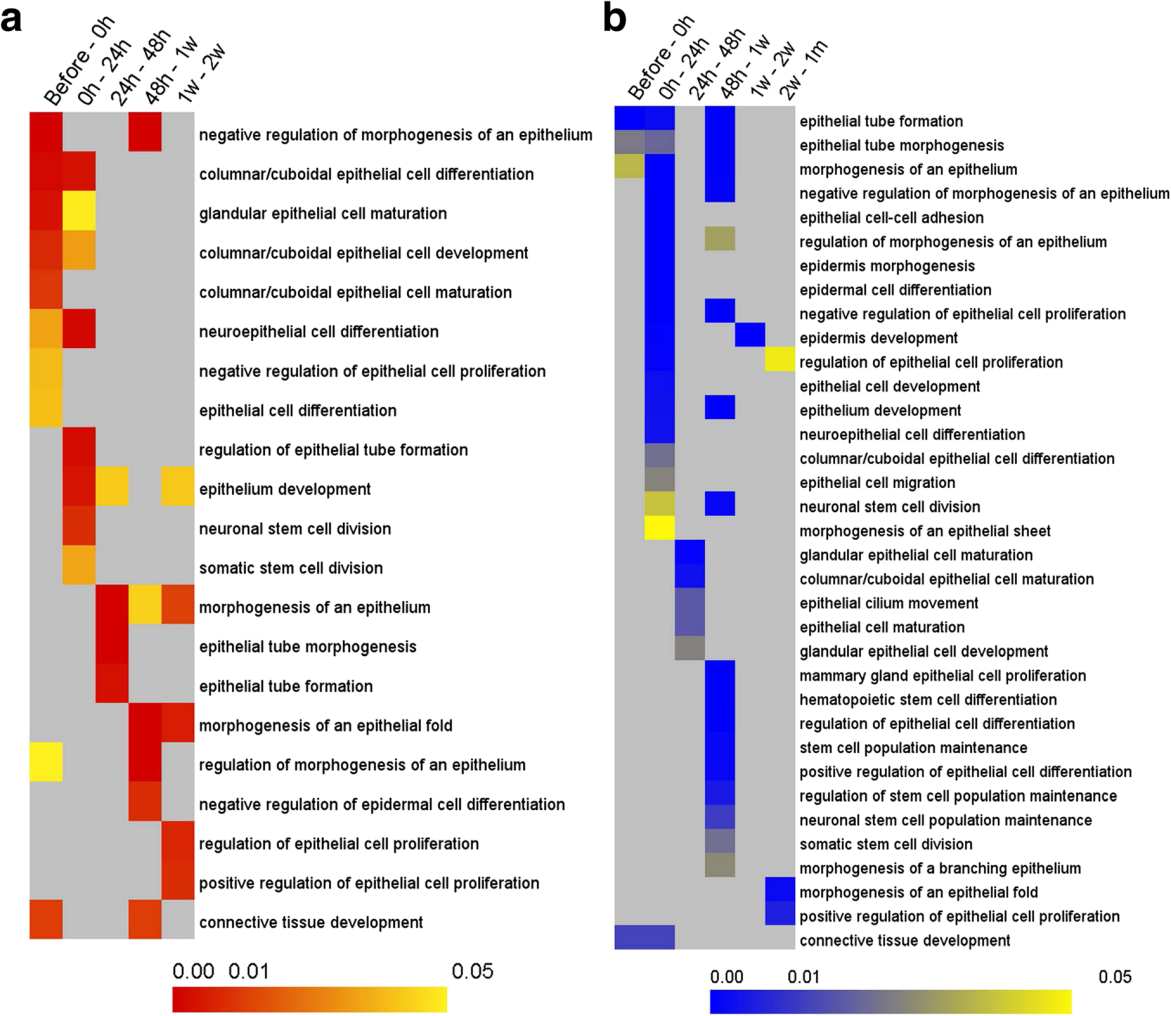
Heat maps of GO enrichment analysis for epithelial cell proliferation and differentiation-related DEGs. **a** Up-regulated DEGs. **b** Down-regulated DEGs. Up or down-regulated DEGs at each time point were submitted to GO enrichment analysis using GOEAST web-based software (http://omicslab.genetics.ac.cn/GOEAST/index.php). The results of GO enrichment analysis are displayed in Additional file 2: Table S2. Biological function in GO terms involved in epithelial cell proliferation and differentiation were selected to display in heat maps according to the statistical significance (*P* < 0.05). Columns and rows in the heat maps indicate treatments and enriched biological process GO terms, respectively. Sample names are displayed above the heat maps. Color scales indicate *P* values of enrichment tests and gray cells represent an empty value or a value > 0.05

In the course of wound healing process, the outer epithelial cells of the mantle graft proliferate and differentiate into pearl sac [38]. So, to know the genes engaged in pearl sac development, we focused on epithelial cell proliferation and differentiation-related GO terms. GO analysis revealed that epithelial cell proliferation and differentiation-related terms were embellished in the biological process category (Additional file 2: Table S2a,b). Among them, we found 21 up-regulated and 35 down-regulated terms relevant to epithelial cell proliferation and differentiation were significantly (*P* < 0.05) enriched during pearl sac formation (before to 2 w) (Fig. 4). It was also observed that epithelial cell proliferation and differentiation-related genes were enriched during the first two weeks of graft transplantation (Fig. 4). After that, there was no significantly up-regulating term pertinent to epithelial cell proliferation (Fig. 4a). Moreover, epithelial cell proliferation related terms were down-regulated after two weeks of grafting (Fig. 4b). These results suggest that the pearl sac formation was completed after two weeks of graft transplantation. The DEGs significantly (*P* < 0.05) up-regulated (34) and down-regulated (88) during the development of pearl sac are listed in Additional file 1: Table S5 and S6.

Epithelial stem cells may be a source of proliferative epithelial cells to form the pearl sac. We observed some stem cell marker genes i.e. *SOX2, MEF2A, HES1, MET, NRP1, ESR1, STAT6, PAX2, FZD1, PROM1* and *ABCG2* that were expressed significantly during the formation of pearl sac (Additional file 1: Table S5).

### Comparison of enrichment studies between DESeq2 and sleuth calculated DEGs

In the first part of the study (KEGG immune pathways and GO cell proliferation), we used the DEGs calculated from DESeq2 [45]. In order to compare the enrichment results, we again calculated DEGs using sleuth [46] to ascertain whether any differences exists in enrichment studies between these two calculations. However, the number of DEGs obtained from sleuth was comparatively lower than that obtained from DESeq2. Also sleuth could not detect any DEGs at 24 h – 48 h, 2 w – 1 m and 1 m – 3 m time combinations. We used both DESeq2 and sleuth estimated DEGs separately for KEGG pathway enrichment analysis. In spite of having variations in the number of DEGs, immune genes were mostly enriched at 0 h – 24 h and 48 h – 1 w time points in both cases (Fig. 3 and Additional file 1: Figure S2). Moreover, there was no apparent changes in the pathways that were significantly up- or down-regulated verifying that the interpretation of our result was not influenced much due to the selection of software like DESeq2 or sleuth.

### Examination of contamination rate of host transcripts to adjust the expression levels of biomineralization-related genes expressed specifically in the pearl sac

In the second part of the study, we focused on biomineralization-related genes specifically expressed in the mantle epithelial cells. These genes are expressed in donor mantle epithelial cells of pearl sac but not in host tissues surrounding the pearl sac. However, as discussed above, our transcriptome data contained transcripts from contaminated host tissues due to the difficulty of separating pearl sac completely from the surrounding host gonad tissues. For the first time, here we estimated the contamination rate based on the calculated donor specific SNV rate (Fig. 1) and then adjusted the expression level (TPM, transcripts per million) of biomineralization-related genes using following equation. After adjusting the expression level, we found that the expression pattern of SMPs is comparable to the previous study which substantiate the potentiality of this method [47].

$$ Adjusted\kern0.1em \mathit{\exp} ression\kern0.3em level= TPM\times \frac{100}{100\kern0.3em -\kern0.3em contamination\kern0.35em rate\kern0.1em \left(\%\right)} $$

Where, *Contamination rate* (%) = 100 − *Donor specific SNV rate* (%)

### Expression profiles of biomineralization-related genes during pearl sac and pearl formation

**Fig. 5:**
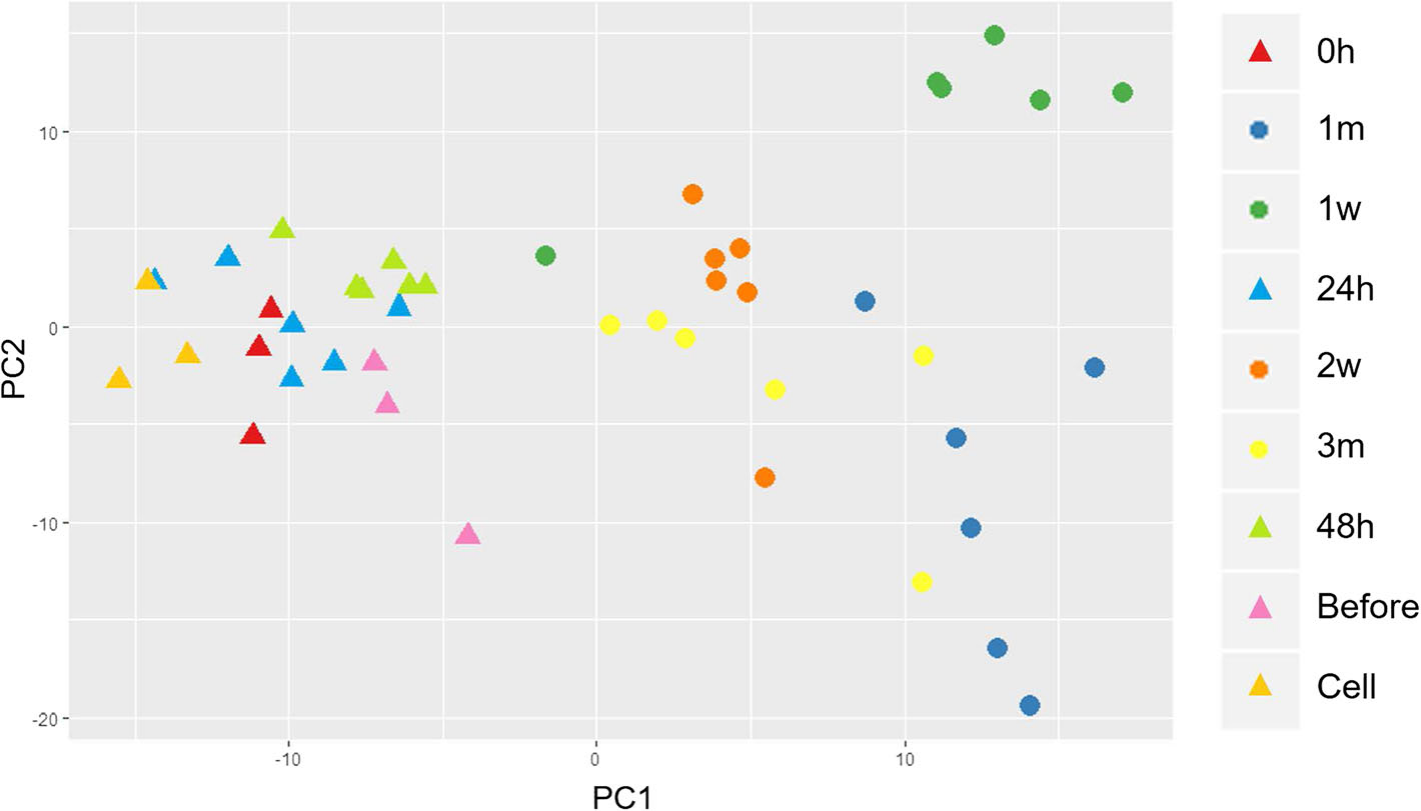
Principle component analysis (PCA) of biomineralization-related gene expression profiles at different phases of pearl grafting. The X- and Y- axes represent PC1 and PC2 respectively. Different colors of data points indicate different time points (cell, before, 0 h, 24 h, 48 h, 1 w, 2 w, 1 m and 3 m). All the samples were clustered in two separate groups as indicated by two different shapes: triangle for cell, before and 0 h to 48 h; round for 1 w to 3 m

**Fig. 6:**
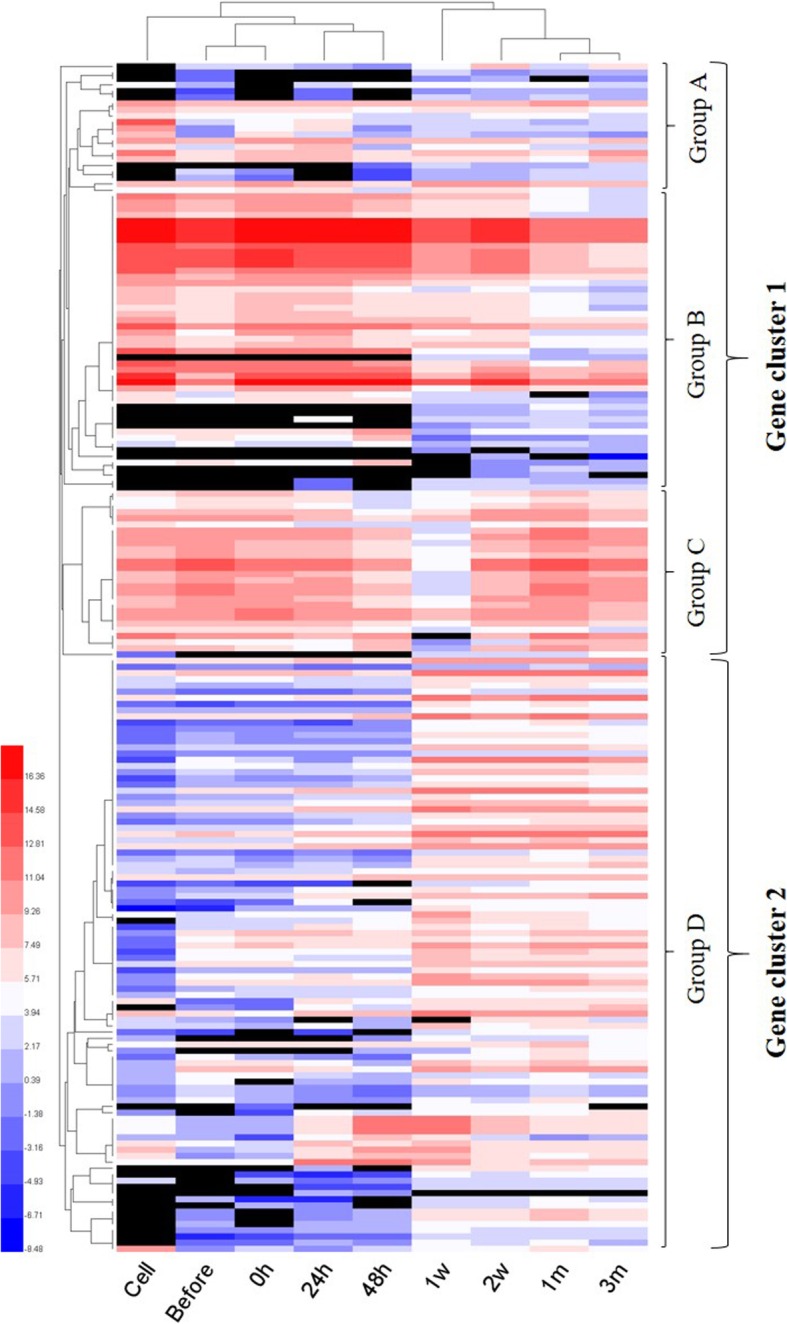
Heat map iillustrating the expression patterns of 192 biomineralization-related genes across various steps of pearl sac and pearl formation. Each column contains the measurements for gene expression change for a single sample. Relative gene expression is indicated by colour: high-expression (red), median-expression (white) and low-expression (blue). Black cells represent very little or no expression. Genes and samples with similar expression profiles are grouped by hierarchical clustering (left and top trees). These 192 genes are listed in Table 2 sequentially

To date, more than 200 molluscan biomineralization-related genes have been identified that contribute to the formation of shell and pearl [44, 48]. Here, we selected 192 biomineralization-related genes from various pearl producing mollusks including *P. fucata* (Additional file 5: Table S7), and our transcripts were annotated to these reference genes. Then the expression levels of all the 192 genes were adjusted according to the above equation. PCA was performed on the 192 biomineralization-related genes. The results of PCA showed clearly different gene expression profiles between earlier (cell, before, 0 h, 24 h and 48 h) and later stages (1 w, 2 w, 1 m and 3 m) after grafting (Fig. 5). Further hierarchical clustering of the 192 genes presented more clear explanation about their expression and contribution during pearl sac and pearl formation (Fig. 6). It was evident that one week after graft transplanting the expression of almost all the genes changed drastically and remained comparable upto the end of three months. In the hierarchical clustering, biomineralization-related genes were separated into four groups named group A – D under two major clusters (Fig. 6). Most of the genes grouped in cluster 1 showed higher expression during the earlier stages of pearl formation followed by a decrease in the later stages, whereas a reverse trend was observed in gene cluster 2. Table 2 listed the genes in different groups and clusters with their recognition in the shell formation.

**Table 2:** Gene clusters obtained from the expression analysis of 192 biomineralization-related genes (genes were listed sequentially from Figure 6)

Gene cluster 1	Group A	ACCBP1^b^, Alkaline phosphatase, ML1A2, Calmodulin^p^, Tyrosinase^p^, 000058 mRNA for hypothetical protein, AP-24^n^, PfTy^p^, PfTy1^b^, KRMP-10^p^, Shematrin-7^b^, Shematrin-5^b^, Shematrin-2beta^b^, Shematrin-3^b^, PFMG5^b^, Prismin-2^p^, C-type lectin 2, Incilarin A, PmCHST11b^n^, PfCB/chitobiase^p^, Tyr-1
	Group B	Aspein^p^, Cement-like protein^p^, SGMP1, PfCN, GRMP^b^, MSI7^b^, Shematrin-2^b^, Shematrin-8^b^, Pmshem-3 for shematrin, Pmshem-2 for shematrin, Shematrin-1^b^, Shematrin-9^b^, PFMG10^b^, MPN88^p^, Prismalin-14^p^, PUSP-20^p^, MSI30/MSI2^p^, shematrin-2alpha^b^, CLP1 protein, MPN^b^, Pfp-16 for hypothetical protein, Pmshem-1 for shematrin, Shematrin-6^b^, Prismin-1^p^, Tyrosinase-2^p^, KRMP-1^p^, Lustrin A^n^, KRMP-3^p^, 000118 mRNA for prism uncharacterized shell protein 18 like, KRMP-2^p^, MSI31^p^, KRMP-7^p^, MPN88-lack6, MPN88-lack7, KRMP-4^p^, Perlucin^n^, Prisilkin-39^p^, Regucalcin, KRMP-8^p^, KRMP-11^p^, N14#3.pro^n^,, 000200 mRNA for hypothetical protein, KRMP-5^p^, KRMP-6^p^, KRMP-9^p^, N16–3/N14#4.pro^n^, Calconectin, C-type lectin 1
	Group C	Chitin binding protein, PfCHS1^n^, N19-2^n^, NSPI-5^n^, 000081 mRNA for Glycine-rich protein 2 like, NUSP-6^n^, Linkine^n^, MSI25 (hypothetical protein), NUSP-3^n^, N19^n^, Pif177^n^, MSI60/insoluble protein^n^, MSI60RP^n^, NUSP-17^n^, N16–1/N14#1.pro^n^, N16-7^n^, Pearlin^n^, MRNP34^n^, MSI80^n^, N36/33, Nacrein^b^, N45, N66^b^, N14#7.pro^n^, N16-6^n^, N16–2/N14#2.pro^n^, Lectin
Gene cluster 2	Group D	AP-1, MSP-1, N151, PfBAMBI, BMP-2, BMP-R2, M45, N44, Pf-POU2F1, Cathepsin B, Dermatopontin^n^, BMP-1B, Calcium/calmodulin-dependent protein kinase I, PfBMPR1B, Homeobox protein-4, BMP-2B, Paramyosin, BMP-7^b^, Calcineurin B subunit, Chitinase 3, L-type voltage-dependent calcium channel beta subunit, Putative uncharacterized protein F18, PfMSX^n^, TFG beta signaling pathway factor, Calreticulin^p^, PfSMAD4, PfDlx^n^, PfYY-1, 67kD laminin receptor precursor, Plasma membrane calcium ATPase, Ferritin-like protein, PfY2, 000031 mRNA for hypothetical protein, 000145 mRNA for hypothetical protein, Chitin synthase 1^b^, SPI (serine proteinase inhibitor), PfSp8-like protein 1, Matrix metalloproteinase, Metallothionein-2, 000194 mRNA for hypothetical protein, SERCA isoform C, Perlwapin-like protein^n^, Pfu000096, BMP-1, SCP-a, SCP-b, Calponin-like protein, Neuronal calcium sensor-1, Calcium-dependent protein kinase, SERCA isoform A, Voltage-dependent L-type calcium channel alpha-1 subunit isoform c, Carbonic anhydrase II, SPARC, Veliger mantle 1, Pf-POU3F4, Metallothionein, PFMG11^b^, PFMG2^b^, N16–5/N14#5.pro^n^, PFMG9^b^, Calmodulin-like protein, BMP-4, Jacalin-related lectin PPL2-a, PFMG3^p^, Carbonic anhydrase precursor, N23^n^, PFMG8, Fam20c^n^, EFCBP, CHST11^n^, PmCHST11a^n^, Incilarin C, EP protein precursor, PfChi1/chitinase 1, PFMG1, PFMG6^b^, PMMG1^n^, PFMG7^b^, PmRunt^n^, PFMG4^b^, PFMG12, Tissue inhibitor of matrix metalloproteinase, AP-7^n^, BMP-3, PfTy2^p^, BMSP, ML1A1, Engrailed, 000066 mRNA for hypothetical protein, Ferritin, ML7A7, Mucoperlin^n^, Perlucin 7^n^, Wnt-1, Wnt-6, Shematrin-4^b^

Different superscript letters indicate genes that are involved in the formation of different shell layers. ^‘p’^: prismatic layer forming gene, ^‘n’^: nacreous layer forming gene and ^‘b’^: both layer forming gene

Shell matrix proteins are secreted from mantle epithelial cells and regulate calcium carbonate crystal formation, resulting in the development of the shell and pearl [4]. Difference in the composition of SMPs is important to determine nacre or prismatic layer characteristics. Therefore, expression patterns of SMPs can be used as a marker of the shell and pearl formation. We investigated the relative expression patterns of 28 representative SMPs from 192 biomineralization-related genes having well-defined implications for quality pearl production that are involved in the formation of prismatic layer (10), nacreous layer (14) and both layer (4) (Table 2). Many of the prismatic layer and both layers forming genes were clumped in the upper part of the gene cluster 1 (group A and B) and exhibited higher expression during the earlier stages (Fig. 6, Table 2). Besides, many nacreous layer forming genes gathered at the lower part of the cluster 1 (group C) and were expressed highly throughout the experiment except in 1 week samples (Fig. 6, Table 2). In cluster 2, most of the genes exhibited lower or no expression during the earlier stages and higher expression in the later stages. Some of the nacreous layer and both layers forming genes were organized in gene cluster 2 (Table 2, Fig. 6).

**Fig. 7:**
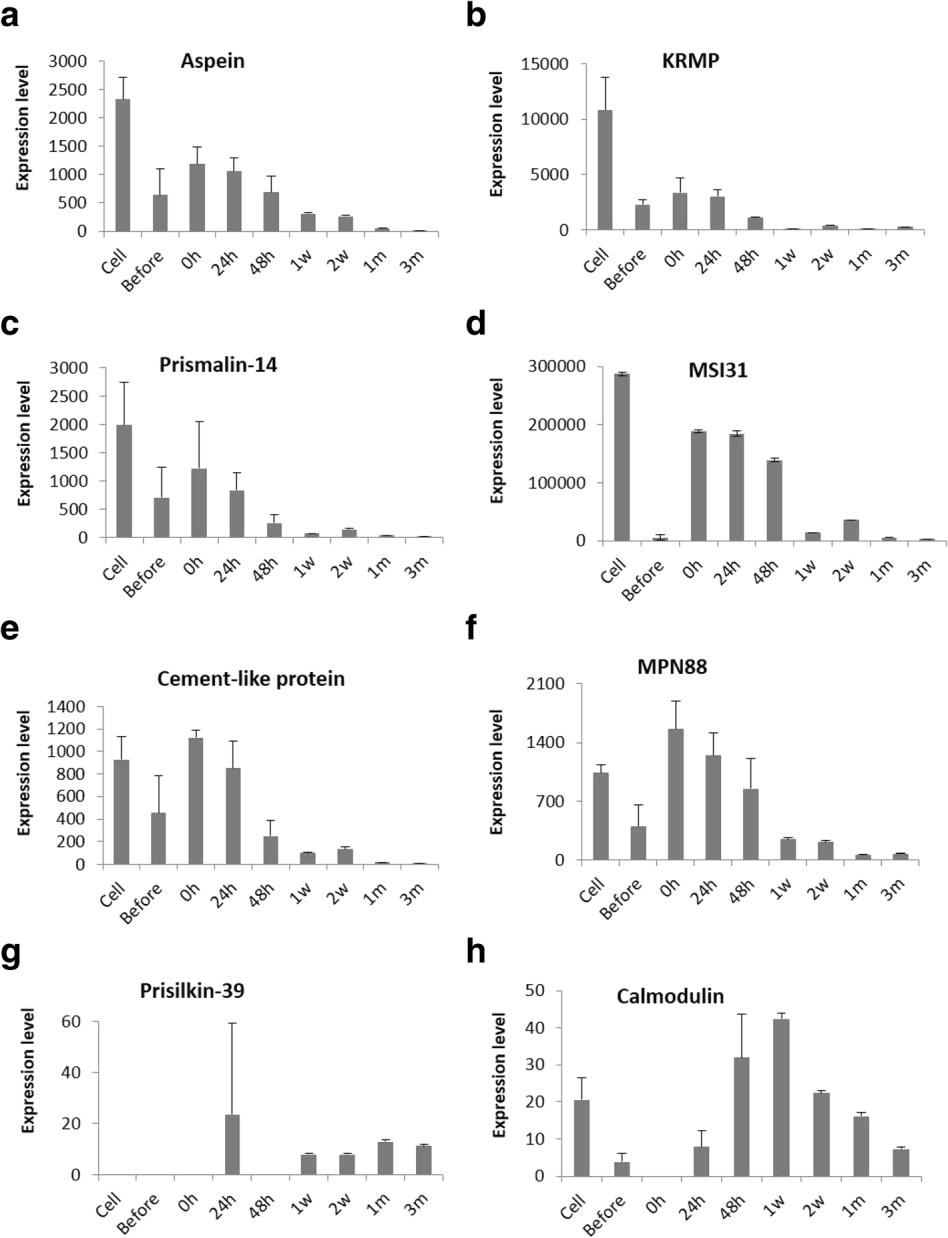
Expression patterns of shell matrix proteins (SMPs) involved in the formation of prismatic layer (**a-h**) at various time points of pearl sac and pearl development. Expression levels are indicated by adjusted TPM values (transcripts per kilobase million)

**Fig. 8:**
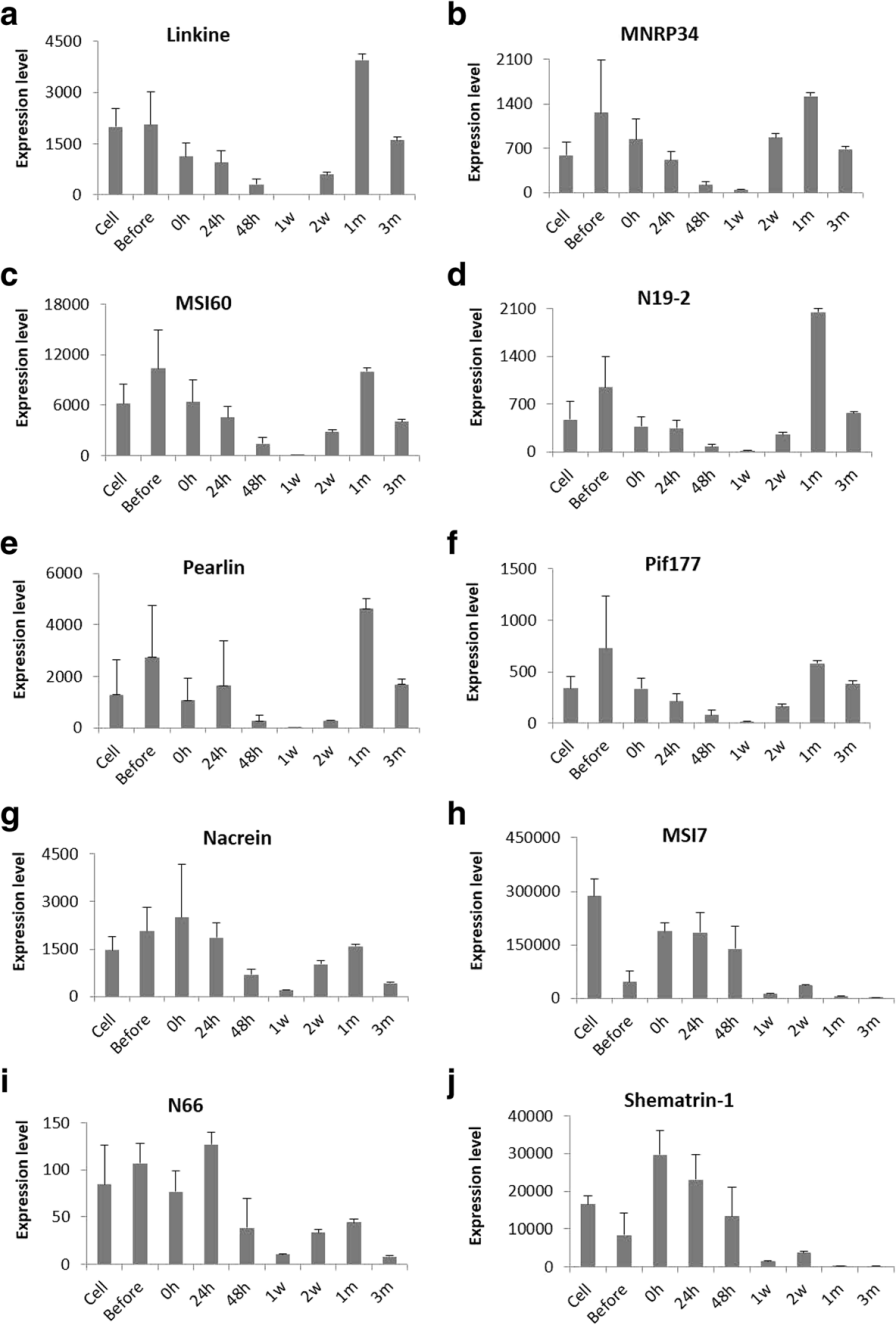
Expression patterns of shell matrix proteins (SMPs) involved in the formation of nacreous layer (**a-f**) and both layer (**g-j**) at various time points of pearl sac and pearl development. Expression levels are indicated by adjusted TPM values (transcripts per kilobase million)

Expression levels of respective SMP genes at different stages are illustrated in Figs. 7 and 8 and Additional file 1: Figure S3. Among the ten prismatic layer forming genes, eight genes were significantly up-regulated during earlier stages and down-regulated in the later stages of pearl development (Fig. 7a-f, Additional file 1: Figure S3a-b); whereas, prisilkin-39 and calmodulin showed different expression patterns from those of other prismatic layer forming genes. In earlier periods, there was little or no expression of prisilkin-39 except the peak at 24 h, then it was expressed increasingly from 1 w to 3 m, showing the second peak at 1 m (Fig. 7g). Calmodulin was observed up-regulating with its highest peak at 1 w before starting to decline to the end (Fig. 7h). KRMP and MSI31 gene expression levels were apparently higher compared to others (Fig. 7b,d).

Most of the nacreous layer forming SMPs showed higher expression before graft transplantation and then down-regulation upto 1 w, after that up-regulation again upto the end with a maximum expression at 1 m (Fig. 8a-f, Additional file 1: Figure S3c-e). Mucoperlin, perlucin-7, perlwapin-like protein, lustrin A and dermatopontin, showed little or no expression since 48 h and then started rising significantly (Additional file 1: Figure S3f-j). Perlucin-7 and perlwapin-like protein reached the maximum expression at 1 w (Additional file 1: Figure S3 g,h), whereas lustrin A and dermatopontin at 2 w and 1 m, respectively (Additional file 1: Figure S3i,j). NUSP-3 and mucoperlin displayed the highest expression at 3 m (Additional file 1: Figure S3e,f). The expression of MSI60 was significantly higher than those of other nacre forming genes (Fig. 8c). Both layers forming genes like nacrein, MSI7, N66 and shematrin-1 showed almost similar trend in expression with many nacreous layer forming genes (Fig. 8g-j). They were up-regulated during earlier periods with a higher expression within 0 h – 24 h, then down-regulated upto 1 w and up-regulated again after 1 w. Expressions of MSI7 and shematrin-1 were relatively higher (Fig. 8h, j).

### Microscopic examination of surface deposition on pearl

**Fig. 9:**
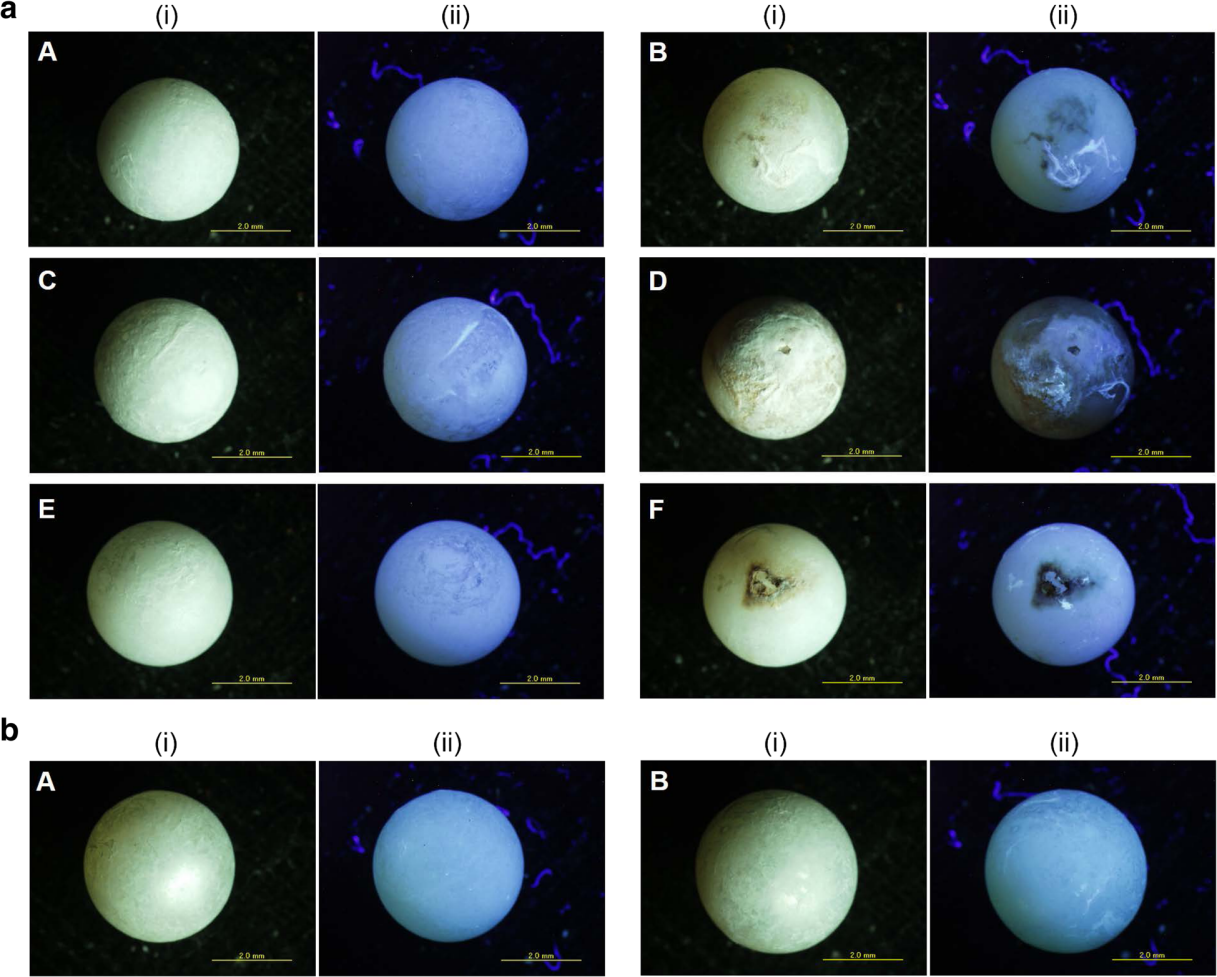
Microscopic images of surface depositions on pearl at (**a**) 1 month and (**b**) 3 months of grafting. (i) Normal microscopic image and (ii) UV fluorescence microscopic image. Uppercase letter A-F indicates different pearls. Scale bars 2.0 mm

Microscopic observations were carried out to scrutinize the internal micro-crystal biomineralization of pearls mediated by the pearl sac epithelium. Surface examination of 1 month pearls revealed the variation in the initial mineralization activity among the pearls (Fig. 9a). Moreover, the irregular surface of pearls illustrated that nacre deposition at the early stage of pearl formation was not uniform throughout the surroundings of a given pearl (Fig. 9a). A visual change on the surface aggregates encircling the nucleus could be noticed at 3 months when the pearl surface became smoother and more regular with a pearl lustre (Fig. 9b).

**Fig. 10:**
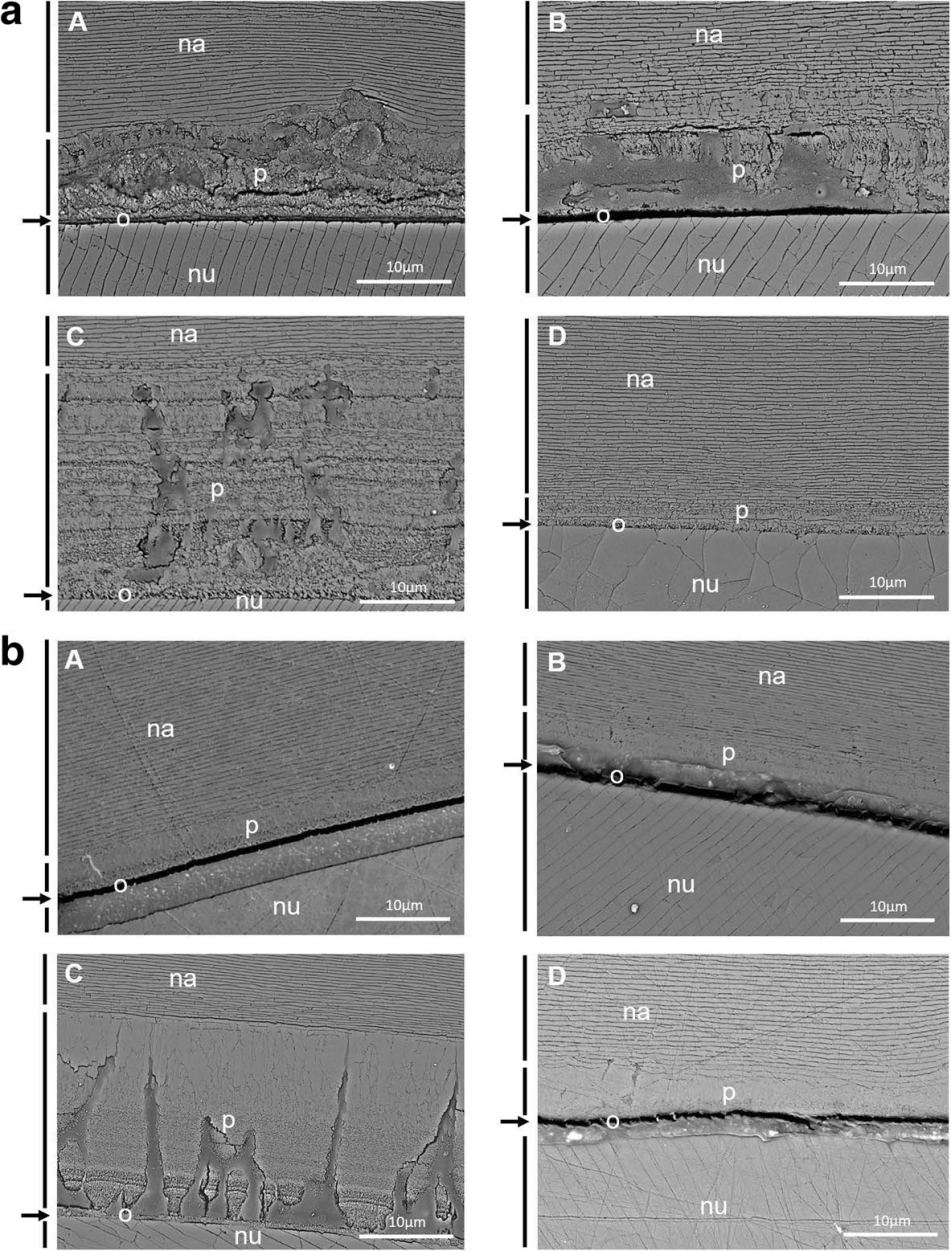
SEM images illustrating microstructural characterization of the pearl layers at (**a**) 1 month and (**b**) 3 months of grafting. nu: nucleus, o: organic layer indicated by black arrow, p: heterogeneous prismatic layer, na: nacreous layer. Uppercase letter A-D indicates different pearls. Scale bars: 10 μm

Scanning electron microscopic (SEM) imaging obtained from the cross section of pearls revealed two distinct pearl layers with clear cut differences in microstructures that were visible on the surface of the nucleus (Fig. 10). Microstructural analysis additionally demonstrated that an initial organic layer was deposited onto the nucleus surface before the secretion of prism and nacre (Fig. 10a, b). It was also noticeable that the thickness of organic material was variable among different pearls and even in different parts of the same pearl (Fig. 10a, b). A heterogeneous prismatic layer in contact with the initial organic layer was then accumulated onto the nucleus before the secretion of the outer aragonite nacreous layer. Unlike mollusk shell, prismatic layer in pearl was diversified. The overall composition of the epithelial secretion during the formation of prismatic layer is variable among the pearls (Fig. 10a, b). There was considerable diversity in the structure of the prismatic layer compared to the regular brick-wall like structures of nacre (Fig. 10a, b). Surface structure of 3 months pearls further suggested the significant increase in nacre in the form of aragonite crystals towards the maturation of the nacreous layer (Fig. 10b).

## Discussion

Recently, RNA-seq, based on next generation sequencing technologies, has become a widely used tool to obtain transcriptomic information on genes of interest that are differentially expressed under certain conditions. In this study, we have generated 925 M sequencing reads from the mantle graft of the pearl oyster, *P. fucata*, and constructed a comprehensive expression profile of genes during the formation of pearl sac and pearl. We identified all the DEGs at different time combinations during grafting and conducted a series of bioinformatic analysis to screen out the key genes and pathways closely related to immune function, epithelial cell proliferation and biomineralization. The highest number of DEGs was obtained during 48 h – 1 w time point. At 48 h after graft implantation many important biological functions like immune reactions, cell proliferations and various metabolic pathways were up-regulated which might be resulted in the maximum number of DEGs assigned at 48 h – 1 w. However, it is very difficult to summarize whether this is because of the up-regulation of many processes or due to the differences in gene expression between donor (48 h) and host (1 w) tissues or both.

### Differentially expressed genes in immune pathways

Graft implantation process causes the oysters stressed. The increased stress during 48 h of graft transplantation causes increased hemocyte infiltration in the wound site [12]. The host oysters showed immune response during the early stages of grafting as indicated by the up-regulation of many immune functions (Fig. 3).

Toll-like receptors (TLRs) are fundamental components of innate immunity playing a significant role in defense against pathogen both in vertebrates [49, 50] and invertebrates like fly [51], oyster [52, 53] and scallop [54, 55]. It is also revealed that many components (TLR, MyD88, TRAF, IKK, NF-κB and so on) in the canonical TLR signaling pathway in the animals from fly to human are rather conserved [55, 56]. Though TLRs mediated innate immune responses both in vertebrates and invertebrates share a common ancient ancestry, the domain organization, mode of activation and functions are diverse [57, 58]. Earlier evidences suggested that, the ancient molluscan TLRs possessed a powerful pattern recognition ability to recognize broader ligands than its mammalian homologues [54, 59], and mediated the downstream signaling cascades in a MyD88-dependent or MyD88-independent pathways to activate the expression of various immune effectors [55, 60–62]. Moreover, two TLRs (ORF06037 and ORF09244) in scallop exhibited a closer phylogenetic relationship to the plasma membrane located TLRs, such as *TLR1*, *TLR2*, *TLR4* and *TLR6* in human and mouse [55]. Among eighty three anticipated TLR genes from Pacific oyster *C. gigas* genome, eighty TLRs are predicted to contain the toll/interleukin-1 receptor (TIR) domain and at least six TLRs have been identified to participate in immune response so far [53, 63]. Thus, higher expressions of TLR signaling pathway at 24 h in our data indicate that host oysters react to the transplanted allograft and induce the immune response (Fig. 3a). Differential expression of TLRs like *TLR1* and *TLR6* and their downstream signaling molecules including TNF-alpha and IL-1 has also been observed previously in hemocytes of *P. fucata* 48 h after the nucleus insertion [33]. Recently, immune function of *TLR6* has also been identified in Pacific oyster *Crassostrea gigas* which exhibits a broader recognition spectrum [59]. Different TLRs enriched in different pathways explain their role in recognizing distinct pathogen-associated molecular profiles (PAMPs) (Additional file 4: Table S4) [59, 64]. *TLR1* and *TLR2* were significantly up-regulated at 0 h, whereas *TLR4* at 48 h, indicating that *TLR4* may play a vital role in wound healing and immune response to the inserted nucleus and mantle graft. In a recent study on *P. fucata*, *TLR4* was also found to increase significantly after implantation peaking at day 2 [65]. *TLR4* is believed to initiate inflammation and tissue injury by responding to both bacterial endotoxin and multiple endogenous ligands, including heat-shock protein (HSP) [66]. Other key molecules of TLR pathway including *NF-κB1* and tumor necrosis factor receptor-associated factor *TRAF2*,*3*,*5* and *6* were also enriched during 0 h – 24 h and 48 h – 1 w in consistence with their role in regulating inflammatory responses (Additional file 4: Table S4). Among seven identified mammalian TRAF family gene (*TRAF1–7*), *TRAF1,2,3* and *7* have already been confirmed to evolve in immune response in oyster [67–71]. The inflammatory functions of *NF-κB1* and TRAF molecules in *P. fucata* were also described earlier [69, 72, 73]. In addition to immune function, NF-κB signaling pathway also involves in shell formation in *P. fucata* by regulating the transcriptional activity of nacrein promoter [74].

A member of caspases, *CASP8*, was activated both in donor mantle graft (before – 0 h and 0 h – 24 h) and host oyster (48 h – 1 w) (Fig. 3a, Additional file 4: Table S4). Caspases are well-known for their important roles in apoptosis [75–77] and inflammation [78, 79]. Thus the early expression of *CASP8* in apoptosis pathway was most likely implicated for cell death in the wounded graft (Fig. 3, Additional file 4: Table S4). The involvement of *CASP8* in other pathways like TLR/NOD-like receptors signaling indicated its non-apoptotic function (Fig. 3, Additional file 4: Table S4). The dual role of *CASP8* in apoptotic and non-apoptotic activity has also been described previously where *CASP8* bears a significant role in cell death as well as in regulating TLR and NF-κB signaling [80]. Moreover, the role of *CASP8* in innate immunity has been described in *C. gigas* against virus [81] and in *C. hongkongenesis* against bacteria [82].

HSPs are the most abundant, ubiquitously expressed, soluble, intracellular proteins and are phylogenetically conserved in all organisms [83]. HSPs are important in regulating the immune responses like activation of macrophages and dendritic cells and in the production of cytokines and chemokines [83, 84]. In this study, several HSPs like *HSP70, HSP71, HSP72, HSP74, HSP83, HSP97* and *HSP7C* were found up-regulated at 48 h – 1 w and down-regulated at 0 h – 24 h, suggesting that HSPs may be induced by surgery and graft transplantation (Additional file 4: Table S4). Host oysters may be more susceptible to the effects of heat or other stress induced by grafting. Recently, the up-regulation of *HSP70* was also detected in *P. fucata* at 0 h – 48 h of allografting [33] and 6 h – 96 h of xenografting [36]. The involvement of HSPs in different immune pathways also conclude their simultaneous role in countering environment stress, immune response, inflammatory process and the regulation of apoptosis.

Mitogen-activated protein kinase (MAPK) signaling pathway enriched in 0 h – 24 h and 48 h – 1 w suggests an important role of this pathway in pearl grafting (Fig. 3a, b). MAPK cascades with conserved function play a critical role in the regulation of many physiological and biochemical processes including cell proliferation, differentiation, cell growth and death, immune reaction, and environmental adaptation [85–87]. In conjunction with the activation of NF-κB and Ras signaling pathway, MAPK activation induces the expression of multiple genes that jointly regulate the inflammatory response [85]. The DEGs in MAPK signaling pathway detected in this study suggest the cooperation of the MAPK signaling pathway in pearl sac and pearl formation. *EGFR* and *FGFR* are important cell surface receptors that can induce MAPK signaling by activating other kinases. Though the predominant function of *EGFR* is related to cell proliferation and differentiation, it also plays an important role in innate immunity in mollusk [88]. Moreover, the up-regulated expression of *EGFR* at 48 h – 1 w and 1 w – 2 w might correlate with wound healing and promotion of cell proliferation and migration (Additional file 4: Table S4) [88]. Signal transduction begins with the activation of small GTPases like RAS and RHO family proteins [89, 90]. Other than the MAPKs, RAS and RHO subfamily proteins were also expressed which have substantial roles in MAPK activation (Additional file 4: Table S4) [90]. Very little is known about the role of MAPKs in pearl oyster. However, some previous studies suggested the involvement of MAPKs in the innate immunity of *C. hongkongenesis* [91, 92]. More recently, a MAP kinase, *MKK4*, was found to be expressed in *P. fucata* 1 day after grafting in response to the nucleus insertion operation indicating its role in host defense mechanism, potentially in protecting the pearl oyster from injury caused by grafting [93].

### Differentially expressed genes during epithelial cells proliferation and differentiation

The mantle tissues of mollusk are metabolically and transcriptionally active and play a pivotal role in shell and pearl biomineralization [94]. After the grafting operation, the donor mantle tissues not only survive but also proliferate to form the pearl sac [7, 12]. Therefore, the genes involved in proliferation and differentiation of outer epithelial cells are of utmost important in the course of pearl formation. Before proliferating into pearl sac, the adhesion between the mantle graft tissues and connective tissues of gonad of the host oysters is a prerequisite that eventually affects the success of nucleus implantation and pearl sac formation [7]. Thus, the proliferation of connective tissue cells among gonadal follicles during pearl sac formation is very significant and found up-regulated at 48 h as indicated by the process of ‘connective tissue development’ [12]. The transplanted grafts were clearly separable at 48 h from the gonad tissues where these were implanted as the development of pearl sac was not initiated by this time [12]. However, after 1 week the transplanted grafts could not be clearly distinguished from the surrounding tissues, indicating that the formation of pearl sac was in progress. The up- and down-regulation of many processes related to epithelial cell proliferation at 1 w – 2 w and 2 w – 1 m, respectively, suggest that the pearl sac formation was completed by 2 weeks. We also observed the most crucial time for pearl sac formation was 1 w – 2 w (Fig. 4). An early report on pearl sac formation in *P. fucata* stated that the bead was completely covered with a monolayer of epithelial cells by day 14 [12] which is in line with the result of the present study. Similarly, pearl-sac formation was observed within 3–7 days after implantation in case of 3 mm nuclei, 4–10 days in the case of 4 mm nuclei and 6–12 days in the case of 5 mm nuclei in *P. fucata* [95]. Two other studies on *P. margaritifera* also showed that the pearl sac development required 12 to 14 days [7, 96]. All of these results indicate the importance of the first two weeks of pearl culture after grafting during which pearl sac is generated.

The differential expression of a number of genes including *JAG1, RFX3, STRC, FGFR2, SAV1, RAC1, DMD, RGMA, PTK7, MAF, MEF2A, SFRP5, TGM1, FZD1, GRHL2, TEAD1, PRKDC, LAMC1, EGFR, CASP8, CDC42, RSPO2, MTSS1, MATN1, SULF1, SPG20* and *LRP6* in some important processes related to epithelial cells proliferation and differentiation rationally indicate their implication in the formation of pearl sac (Additional file 1: Table S5). So far we know, the function of these genes has not been described yet in mollusk except epidermal growth factor receptor (*EGFR*). Being a member of the epidermal growth factor family, *EGFR* primarily functions in development, growth and tissue regeneration [97]. *EGFR* was found to be expressed specifically in the mantle and the pearl sac of *P. fucata*, interpreting its possible role in pearl formation [98]. In the present study, *EGFR* was up-regulated during 48 h – 1 w and 1 w – 2 w time points and then down-regulated at 2 w – 1 m, which definitely clarifies its role in the development of pearl sac (Additional file 1: Table S5).

Though there is no very substantiating evidence about the stem cells that contribute to pearl sac development, but the outer epithelia in the central zone display the characteristic features of the stem cell, i.e. high proliferation rate and high content of saccharides [99]. Here, we determined several stem cell marker genes of which *ABCG2, FZD1, HES1, MEF2A* and *ESR1* were up-regulated and *MET, NRP1, STAT6, PAX2, PROM1, ESR1* and *SOX2* were down-regulated (Additional file 1: Table S5). The differential expression of these genes during the first two weeks of pearl culture definitely suggests that the outer epithelium possesses stem cells which proliferate into pearl sac. Besides, the enrichment of these genes in epithelial cell proliferation and differentiation-related processes clarify their potentiality in the formation of pearl sac. Stem cell-specific transcription factor *SOX2* was also very recently identified from proliferating gonad duct of Pacific oyster [100]. The signal transducers and activators of the transcription family gene *STAT* have been reported for *P. fucata* in a previous study, where it was up-regulated upon grafting peaking at day 3 [32]. *STAT* is stimulated by nucleus grafting operation and may have different functions, including wound repair and the immune response to the graft [32].

### Gene expression profiles during the formation of pearl sac and pearl

**Fig. 11:**
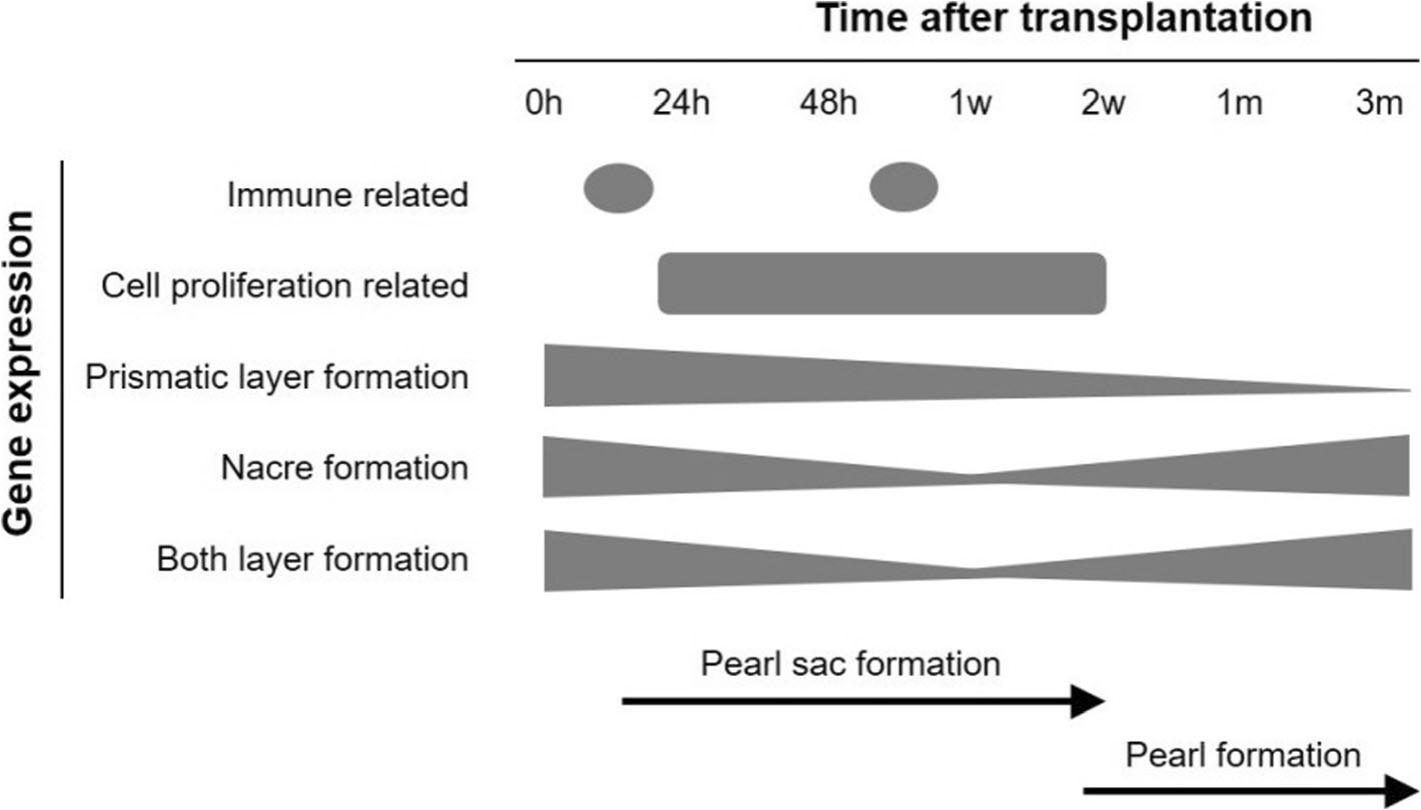
Summary of the study showing gene expression pattern during the pearl sac and pearl formation

The gene expression profiling describes clearly distinct pattern between earlier and later stages of pearl formation (Fig. 5). During the preliminary stages of pearl grafting immune and cell proliferation related-genes were mostly enriched whereas in later stages biomineralization genes (Fig. 11). Shell or pearl biomineralization is a complex process that is strictly controlled by the cascades of a considerable number of genes. Though the mantle tissue of mollusk is primarily responsible for shell biomineralization, it has also been reported that oyster hemocytes can mediate shell biomineralization by binding calcium ion as well as forming CaCO_3_ crystals [101–103]. More recently, some studies also concluded that in addition to mineral transportation, hemocytes contribute to the secretion of the extracellular matrix required for shell biomineralization in bivalve [104, 105]. Therefore, the interaction between the epithelial cells of donor mantle and host hemocytes is very essential for the proper development of pearl sac and pearl [38]. However, the specific role of hemocytes in pearl biomineralization is still obscure.

It has already been clarified that the biomineralization genes are being expressed by the pearl sac developed from donor mantle graft [44, 106–108]. The identified biomineralization-related genes in this study were expressed in the pearl sac, i.e. in the donor mantle cells. Hierarchical clustering considering only biomineralization-related transcripts precisely deciphers that the mineralization process during the first 3 months of culture is regulated differently (Fig. 6). All along the first 2 days after transplantation gene expression remains more or less constant followed by a drastic change after 1 week either positively or negatively. Most of the prismatic layer forming genes showed higher expression in earlier stages, whereas nacreous layer forming genes were mainly enriched in later stages (Fig. 6). This result recapitulates the mineralization sequence, where prismatic layer is secreted first and followed by nacreous layer. From the SEM imaging of pearls, it is also worth noticing that the initial crystal development contains heterogeneous prism and organic material compared to the regular nacre structure that develops later on it (Fig. 10). The inflammatory reaction of host oyster to the transplanted graft sometimes causes heavy accumulation of hemocytes in the wound sites leading to the undesirable secretion of organic layers on the pearl surface [38]. This organic layer is comparable to the periostracum layer of the shell which acts as a basis for the secretion of prism and nacre [2, 38, 109, 110]. Some previous studies also enlightened that the first deposition on the nucleus is aragonitic [47, 110] or calcitic [111] prismatic layer in association with the organic layer detected from the cross section of the pearl. However, the prismatic layer surrounding the pearl is more complex than that of mollusk shell as it contains both aragonite and calcite crystals secreted simultaneously by the pearl sac epithelium [109, 110]. The expression pattern of major SMPs also revealed the concurrent release of aragonite and calcite during the early stage of mineralization (Figs. 7 and 8). The nacreous layer then forms on the top of the prismatic layer [47].

SMPs are considered to play an important role in crystal nucleation, crystal growth and inhibition, crystal polymorphism, crystal morphology, and atomic lattice orientation [47]. They act as a basis for the quality of pearl formation. But the fate of the SMPs during pearl development still needs to be exemplified. It is notable that during shell biomineralization prisms and nacre are assembled from very different protein repertories while in pearl biomineralization the same cells secrete both prism and nacre [22]. Each of the SMPs evolves a specific function in constructing the shell/pearl microstructure either in the form of prism or nacre. Aspein is involved in prismatic calcite formation [18, 19], while the framework protein prismalin-14 mediates chitin and calcium carbonate crystals [20]. Another framework protein shematrin facilitates calcification of the prismatic microstructure [29], whereas MSI7 inhibits the calcite formation [28]. An acidic matrix protein, pif, can induce the nucleation of aragonite crystals and has been reported to regulate the formation of nacreous layer [23]. MSI60 with several characteristic domains constitutes the baseline of the nacreous layer [14, 112]. Pearlin, after being fixed to substrate, induces the formation of aragonite crystals [15]. It has been reported that both N16 and N19 can inhibit the crystallization of calcite and therefore are essential to modify the morphology of CaCO3 crystals and orient nacre growth [25, 26, 113].

The relative expression levels of SMPs seem crucial in controlling the quality of the pearl. Most of the studied prismatic layer forming genes including aspein, KRMP, prismalin-14, MSI31, cement-like protein, MPN88 and PUSP-20 were highly expressed before the maturation of pearl sac, but the level of expression decreased with time of culture (Fig. 7a-f, Additional file 1: Figure S3a). After the complete maturation of pearl sac, their expression levels were relatively low. Presumably, it takes 15 to 20 days to complete the formation of the prismatic layer around the nucleus until when the nacreous layer formation was not started yet [47]. Compared to others prisilkin-39, calmodulin and prismin were expressed higher even after the formation of pearl sac, possibly indicating their roles in the regulation of crystal growth (Fig. 7g, h, Additional file 1: Figure S3b). Prisilkin-39 showed a similar pattern of expression with a previous shell notching experiment where the first peak was observed on day 2 before start decreasing followed by an increase on day 7 (Fig. 7g) [114]. Having the dual function, prisilkin-39 is involved both in constructing the chitinous framework and in regulating the crystal growth during the prismatic layer mineralization [21]. Moreover, the up-regulation of these prismatic layer forming genes during the earlier stages of pearl formation indicates their possible contribution to the development of pearl sac. In a recent study on freshwater pearl mussel *Hyriopsis schlegelii*, calmodulin was found significantly up-regulated during pearl sac formation, suggesting that it might facilitate pearl sac formation [115]. Similarly, we also found that calmodulin was highly up-regulated during pearl sac formation in *P. fucata*, suggesting their potential role in the development of pearl sac (Fig. 7h).

On the other hand, nacreous layer and both layers forming genes were down-regulated during the formation of pearl sac (Fig. 8, Additional file 1: Figure S3c-j). However, the increased expression of nacre forming genes immediately after grafting is because the grafts were prepared from nacre secreting mantle. Thus, it is expected to be a simple continuation of the mineralizing activity of the graft. After the maturation of pearl sac, nacre forming genes were up-regulated with the highest expression during 1 m (Fig. 8a-f), suggesting the accomplishment of nacreous layer formation. Microstructural observation of surface deposition obtained from the pearls at 1 month also indicated the accumulation of significant amount of nacre confirming the deposition of nacreous layer encircling the nucleus (Figs. 9a and 10a). A previous study explained that the nacreous layer formed on the nucleus 35 days after grafting [47]. However, nacreous deposition is not linear [116] throughout the pearl formation process and the highest deposition rate was observed during the first 3 months of culture (Figs. 9b and 10b) [116]. In a prior study on the expression of MSI60, N19, N16, Pif80 and nacrein in pearl sac, the highest expression was detected on day 25, whereas the expression was relatively lower between 15 and 25 days, indicating their involvement in the appearance of the round flat tablets during pearl formation in *P. fucata* [47]. In another study from 3 months to 9 months of culture, the relative expressions of Pif, MSI60 and pearlin were significantly higher at 3 months culture than at 6 or 9 months [117]. The increase or decrease in gene expression may be linked to the calcification rate, which marks their contribution to the gradual formation of the nacreous layer surrounding the nucleus. Nacre weight and thickness is significantly correlated with pearlin, Pif177 and MSI60 gene expression levels [117]. On the other hand, the low expression of genes like aspein and shematrin can result in a top quality pearl by inhibiting the prismatic layer formation [117].

## Conclusions

The findings in the present study conclude two consecutive stages during the 3 months of pearl culture, one is the initiation of pearl sac formation as a part of wound healing process in response to oyster defense mechanism (before 1 week). Another is the maturation of pearl sac and the deposition of organic matrixes on the bare nucleus (1 week to 3 months). The results provide insight into the increased understanding of the immune reaction of the host oyster in response to accepting a transplant. The study also gives some valuable information for identifying the functional genes involved in the formation of pearl sac. The expression profiling of 192 biomineralization genes indicates that first 3 months of pearl biogenesis is very crucial when the pearl sac forms and secretes significant amount of nacre for making a lustrous pearl. Gene expression as well as the microstructural characterization of pearls explain the order of mineralization where a heterogeneous prismatic layer is deposited first onto the nucleus and followed by aragonitic nacreous layer. The improved understanding of the molecular mechanism underlying pearl formation obtained from this study will provide a basis for future research towards upgrading the pearl culture practice and pearl quality.

## Methods

### Experimental animal and mantle grafting

About 2 years old healthy pearl oysters, *P. fucata*, were used as donor and recipient for mantle grafting experiment. Mantle was dissected out from three donor oysters and a strip of mantle tissue was excised from mantle pallium for graft preparation and transplanted into 42 recipient oysters. Graft transplantation was performed by a skilled technician at the Mikimoto pearl farm, Mie, Japan. Two host oysters for each sampling received graft from the same donor (Additional file 1: Figure S4a). During grafting experiments for three months, we collected nine samples i.e. donor mantle epithelial cells (cell), donor mantle pallium (before), donor mantle pallium on grafting but before transplantation (0 h), 24 h, 48 h, 1 w, 2 w, 1 m, and 3 m post grafting) (Additional file 1: Figure S4b-c) and preserved in RNAlater® solution (Ambion, USA) at ˗80 °C until RNA extraction. Mantle epithelial cells were separated as described by Awaji and Machi [38]. Pearl sacs at 1 w, 2 w, 1 m and 3 m were contaminated with host tissues due to the difficulty of separating pearl sac completely from the surrounding host gonad tissues.

### RNA extraction and cDNA library preparation

Total RNA was extracted from the RNA later preserved samples with the RNeasy Mini Kit (QIAGEN, Hilden, Germany) according to the manufacturer’s instructions. RNA quality and integrity were assessed on an Agilent 2200 Tapestation (Agilent Technologies, CA, USA) using RNA ScreenTape. RNA concentrations were measured by Qubit® 2.0 Fluorometer RNA assay kit (Life Technologies, CA, USA).

A total of 2 μg RNA per sample was used as input materials for mRNA sample preparations. Sequencing libraries were generated using the TruSeq Stranded mRNA Library Prep Kit (Illumina, USA) following the manufacturer’s recommendations. Briefly, mRNA was purified from total RNA and fragmented before first strand and second strand cDNA syntheses. The first-strand cDNA was synthetized with the mRNA fragments using SuperScript II Reverse Transcriptase and random hexamer primers. Then the second-strand cDNA was synthetized using DNA polymerase I. Index adapters were then added to identify sequences for each sample in the final data. The quality of the libraries was assessed on the Agilent Bioanalyzer 2200 system. Finally, the libraries were paired-end sequenced on an Illumina Hiseq 4000 platform at BGI, Japan, and 100 bp paired-end reads were generated.

### RNA-seq data analysis

Raw sequences were transformed into clean reads after removing the adapter sequences and low-quality reads (Q < 20). The resulting clean reads were then de novo assembled using Trinity version 2.4.0 with standard settings [118] and pseudo-aligned to the reference *P. fucata* genome using Kallisto [43]. Assembled contigs were annotated by Trinotate for a BLAST search against the Swiss-Prot, RNAMMER, GO, COG, Pfam, and KEGG, and by in-house script for a BLAST search against NCBI NT. The quantified reads were then used to determine the differential gene expression of 2 groups of samples with a threshold criteria FDR 0.01 and log2 ratio. Statistical analysis software R was used for preprocessing and the bioconductor package DESeq2 [45] and sleuth [46] were used for differential gene expression analysis of RNAseq data. The total up- and down-regulated DEGs at seven time combinations (before – 0 h, 0 h – 24 h, 24 h – 48 h, 48 h – 1 w, 1 w – 2 w, 2 w – 1 m and 1 m – 3 m) were used for subsequent GO and KEGG pathway enrichment analyses.

The GO annotations were functionally classified by GOEAST web-based software (http://omicslab.genetics.ac.cn/GOEAST/index.php) [119] for gene function distributions and the GO terms with a corrected *P* -value < 0.05 were considered significantly enriched by the differentially expressed genes. KOBAS 3.0 software was used to screen out the differentially expressed immune genes statistically enriched (*P* < 0.05) in KEGG immune pathways [120]. The hypergeometric test was used to identify the significant KEGG pathways and the *P*-value was corrected by the Benjamini and Hochberg method. Biomineralization-related transcripts were screened by searching BLAST (blastn for similar species and tblastx for different species) using CLC Genomics Workbench against a list of reference biomineralization genes prepared beforehand. The transcript with the lowest E-value and the highest bit score was identified as the best homolog of the reference gene. The expression level of gene was represented as transcripts per million.

### Estimation of host contamination rate

In order to calculate donor specific SNVs, RNA-seq sequencing data were mapped with STAR (version 2.5.3a) using 2-pass mapping [121]. HaplotypeCaller module of Genome Analysis Toolkit (version 3.8–0) was used to call SNV and SNVs with the genotype quality less than 10 were removed [122]. Then contamination ratio was estimated for each donor based on the sample of 0 h as a reference. For example, in the case of donor A, 0 h donor A sample was compared with all sample data not containing donor A, and donor A specific SNVs that appeared only in 0 h donor A were extracted. The number of reads with donor A specific SNV and the number of other reads were added each other and the percentage of reads with donor A specific SNV at 0 h donor A was taken as 100%. Except 0 h, for the other donor A samples, the contamination rate of the host was calculated by the ratio of the donor A specific SNV reads to 0 h.

### Microscopic observation of pearls

The surface depositions of the obtained pearls were observed using an optical microscope VHX-700F (Keyence) at Mikimoto pearl research laboratory. For SEM, the pearls were cut in half by ISOMET diamond cutter (BUEHLER) and embedded in Resin. Surface of embedded samples were polished with ECOMET polisher (BUEHLER) and aluminum oxide. After etching by NaOH, transverse sections of pearls were examined using a scanning electron microscope SU3500 (Hitachi) at Mikimoto Pharmaceutical Co. Ltd.

## Additional files

Additional file 1: **Table S1.** Statistical analysis of transcriptome sequencing data for each sample. **Table S5.** Genes significantly (*P* < 0.05) enriched in epithelial cell proliferation and differentiation related GO terms. **Table S6.** Gene symbol with full gene name. **Figure S1.** Heat map demonstrating whole gene expression profile at different stages of pearl grafting. **Figure S2.** Heat maps of KEGG pathway enrichment analysis for immune related DEGs estimated by sleuth. (a) Up-regulated DEGs. (b) Down-regulated DEGs. **Figure S3.** Expression patterns of SMPs involved in prismatic layer (a-b) and nacreous layer formation (c-j) at various time points of pearl sac and pearl development. **Figure S4.** Experimental design for mantle grafting. (a) donor and host oysters used for grafting, (b) grafting process, and (c) sampling schedule. (PDF 2403 kb)

Additional file 2: **Table S2.** GO analysis. Summary of three functional GO categories for up- and down-regulated DEGs at different time points. (XLSX 257 kb)

Additional file 3: **Table S3.** GO enrichment analysis for immune-related DEGs at 0 h – 24 h and 48 h – 1 w time points. (XLSX 39 kb)

Additional file 4: **Table S4.** Up- and down-regulated DEGs involved in KEGG immune pathways. (XLSX 12 kb)

Additional file 5: **Table S7.** Details of 192 biomineralization-related genes. (XLSX 26 kb)

## Abbreviations

DEGs: Differentially expressed genes
EGFR: Epidermal growth factor receptor
GO: Gene ontology
HSPs: Heat shock proteins
KEGG: Kyoto encyclopedia of genes and genomes
MAPK: Mitogen-activated protein kinase
PAMPs: Pathogen-associated molecular profiles
PCA: Principal component analysis
SEM: Scanning electron microscope
SMPs: Shell matrix proteins
SNVs: Single nucleotide variants
TLRs: Toll-like receptors
TPM: Transcripts per million
