# Supplementary material for: Gene expression profiles at different stages for formation of pearl sac and pearl in the pearl oyster Pinctada fucata
Source: BMC Genomics. 2019 Mar 25;20:240. doi: 10.1186/s12864-019-5579-3 (PMC6434816; doi:10.1186/s12864-019-5579-3)
Supplement: Supplementary file 1 — Table S1. Statistical analysis of transcriptome sequencing data for each sample. Table S5. Genes significantly (P < 0.05) enriched in epithelial cell proliferation and differentiation related GO terms. Table S6. Gene symbol with full gene name. Figure S1. Heat map demonstrating whole gene expression profile at different stages of pearl grafting. Figure S2. Heat maps of KEGG pathway enrichment analysis for immune related DEGs estimated by sleuth. (a) Up-regulated DEGs. (b) Down-regulated DEGs. Figure S3. Expression patterns of SMPs involved in prismatic layer (a-b) and nacreous layer formation (c-j) at various time points of pearl sac and pearl development. Figure S4. Experimental design for mantle grafting. (a) donor and host oysters used for grafting, (b) grafting process, and (c) sampling schedule. (PDF 2403 kb) [file 12864_2019_5579_MOESM1_ESM.pdf]

Gene expression profiles at different stages for formation of pearl sac and pearl in the pearl oyster *Pinctada fucata*

Mariom[1,2], Saori Take[1], Yoji Igarashi[1], Kazutoshi Yoshitake[1], Shuichi Asakawa[1], Kaoru Maeyama[3], Kiyohito Nagai[4], Shugo Watabe[5], Shigeharu Kinoshita[1*]

[1]Graduate School of Agricultural and Life Sciences, The University of Tokyo, Bunkyo, Tokyo 113-8657, Japan

[2]Department of Fisheries Biology and Genetics, Faculty of Fisheries, Bangladesh Agricultural University, Mymensingh-2202, Bangladesh

[3]Mikimoto Pharmaceutical CO., LTD., Kurose 1425, Ise, Mie 516-8581, Japan

[4]Pearl Research Laboratory, K. MIKIMOTO & CO., LTD., Osaki Hazako 923, Hamajima, Shima, Mie 517-0403, Japan

[5]School of Marine Biosciences, Kitasato University, Minami, Sagamihara, Kanagawa 252-0313, Japan

*Correspondence: akino@mail.ecc.u-tokyo.ac.jp

Co-authors email address:
mariom.bau@gmail.com, tksor0523@gmail.com, aiga@mail.ecc.u-tokyo.ac.jp, akyoshita@g.ecc.u-tokyo.ac.jp, asakawa@mail.ecc.u-tokyo.ac.jp, maeyama.511@mikimoto-cosme.com, k-nagai@mikimoto.com, swatabe@kitasato-u.ac.jp

Table S1. Statistical analysis of transcriptome sequencing data for each sample

Sample Name	Clean Reads	Clean bases	Read length (bp)	Q20 (%)	GC content	Quantified Reads ($\times 10^6$)	Quantification ratio (%)
Before_A	17,164,384	1,716,438,400	100	98.32	43.20%	16.73	67.75
Before_B	10,026,656	1,002,665,600	100	98.20	43.32%	10.15	68.77
Before_C	12,059,064	1,205,906,400	100	98.24	42.67%	12.18	68.50
0h_A	9,811,942	981,194,200	100	98.17	44.64%	9.74	68.34
0h_B	9,412,514	941,251,400	100	98.22	45.18%	9.27	68.14
0h_C	10,398,038	1,039,803,800	100	98.40	44.86%	11.02	70.54
24h_A1	10,355,918	1,035,591,800	100	98.20	43.00%	10.24	68.09
24h_A2	7,867,464	786,746,400	100	98.44	43.25%	8.08	71.73
24h_B1	8,870,314	887,031,400	100	98.61	42.38%	8.73	72.15
24h_B2	7,403,436	740,343,600	100	98.63	43.34%	22.58	67.36
24h_C1	23,143,452	2,314,345,200	100	98.23	40.50%	9.53	71.33
24h_C2	12,869,554	1,286,955,400	100	98.05	41.23%	13.01	68.30
48h_A1	7,112,274	711,227,400	100	98.62	42.38%	7.39	69.98
48h_A2	6,976,730	697,673,000	100	98.50	41.03%	9.48	68.07
48h_B1	9,891,620	989,162,000	100	98.42	40.95%	6.99	68.82
48h_B2	9,625,184	962,518,400	100	98.35	42.74%	9.19	69.26
48h_C1	9,008,866	900,886,600	100	98.33	41.43%	9.82	68.42
48h_C2	5,665,372	566,537,200	100	98.69	42.34%	5.97	70.70
1w_A1	48,419,298	4,841,929,800	100	95.58	41.15%	40.27	61.44
1w_A2	23,539,310	2,353,931,000	100	96.39	41.38%	21.27	64.77
1w_B1	36,485,718	3,648,571,800	100	96.32	41.45%	32.36	64.06
1w_B2	17,106,708	1,710,670,800	100	95.67	44.09%	14.51	64.41
1w_C1	5,467,236	546,723,600	100	97.90	50.37%	3.83	64.10
1w_C2	30,277,428	3,027,742,800	100	96.51	41.59%	27.64	64.71
2w_A1	34,149,422	3,414,942,200	100	96.46	41.18%	28.76	62.37
2w_A2	69,600,066	6,960,006,600	100	96.03	40.42%	61.38	63.54
2w_B1	32,927,364	3,292,736,400	100	97.05	41.53%	29.5	64.44
2w_B2	26,223,104	2,622,310,400	100	96.49	40.58%	22.49	63.11
2w_C1	38,238,402	3,823,840,200	100	96.50	42.09%	31.83	62.69
2w_C2	55,393,672	5,539,367,200	100	95.13	41.81%	45.3	62.51
1m_A1	45,558,068	4,555,806,800	100	95.67	40.62%	39.64	62.91
1m_A2	26,248,864	2,624,886,400	100	97.31	41.29%	22.59	63.00
1m_B1	25,934,080	2,593,408,000	100	96.55	40.29%	21.51	61.10
1m_B2	32,196,990	3,219,699,000	100	96.97	44.01%	25.97	62.89
1m_C1	25,541,512	2,554,151,200	100	96.38	40.22%	19.85	58.60
1m_C2	32,021,402	3,202,140,200	100	96.35	40.85%	26.87	61.33
3m_A1	23,359,108	2,335,910,800	100	95.64	40.84%	19.25	60.50
3m_A2	24,171,408	2,417,140,800	100	96.33	42.24%	18.64	58.99
3m_B1	30,337,036	3,033,703,600	100	96.66	41.62%	23.83	60.16
3m_B2	11,595,252	1,159,525,200	100	96.56	40.22%	8.99	58.99
3m_C1	1,231,312	123,131,200	100	97.30	39.04%	1.2	66.43
3m_C2	22,436,394	2,243,639,400	100	96.39	40.34%	17.84	59.91
Cell_A	7,382,306	738,230,600	100	98.34	44.65%	7.1	68.67
Cell_B	5,825,620	582,562,000	100	98.26	44.03%	5.37	66.89
Cell_C	6,019,878	601,987,800	100	98.48	45.08%	5.88	69.79

Table S5. Genes significantly ($P < 0.05$) enriched in epithelial cell proliferation and differentiation related GO terms

GO term	Up-regulated DEGs	Down-regulated DEGs
negative regulation of morphogenesis of an epithelium	*SULF1*	*SULF1, LRP6*
columnar/cuboidal epithelial cell differentiation	*JAG1, RFX3, STRC*	*JAG1, FGFR2, SAV1, RAC1*
glandular epithelial cell maturation	*RFX3*	*RFX3*
columnar/cuboidal epithelial cell development	*RFX3, STRC*	-
columnar/cuboidal epithelial cell maturation	*RFX3*	*RFX3*
neuro-epithelial cell differentiation	*STRC, JAG1*	*JAG1, RAC1*
negative regulation of epithelial cell proliferation	*TSC2, FBXW7, PTPRK, SULF1, MTSS1*	*PTPRK, TSC2, FBXW7, MTSS1, SULF1, PTPRM, LRP6, SAV1, XDH*
epithelial cell differentiation	*EHF, TGM1, RFX3, STRC, JAG1, MTSS1, MYO1E*	-
regulation of epithelial tube formation	*PTK7, SFRP5, FZD1, LRP6*	-
epithelium development	*RFX3, DMD, RGMA, PTK7, MAF, MEF2A, SFRP5, TGM1, FZD1, GRHL2, TEAD1, PRKDC*	*TSC2, EHF, RUNX1, JAG1, RAB10, FGFR2, EP300, ENAH, CASP8, CDC42, MTSS1, LRP4, MMP12, LRP6, NRP1, ILK, IPMK, PAK1, SAV1, CLIC4, RAC1, MYO1E, EGFR, TBX1, LBX1*
neuronal stem cell division	*FGFR1, LRP6*	*RAB10, FGFR2, LRP6*
somatic stem cell division	*FGFR1, LRP6*	*LRP6, FGFR2, ASPM, DOCK7*
morphogenesis of an epithelium	*CASP8, CDC42, RSPO2, MTSS1*	*TBX1, RAB10, CASP8*
epithelial tube morphogenesis	*CASP8, RSPO2, MTSS1*	*TBX1, CASP8*
epithelial tube formation	*CASP8*	*CASP8*
morphogenesis of an epithelial fold	*EGFR, AR*	*EGFR*
regulation of morphogenesis of an epithelium	*SFRP5, WNT2B, LRP6, AR, SULF1, ESR1, RAC1*	*FGFR2, SULF1, LRP6, RAC1*
negative regulation of epidermal cell differentiation	*MSX2, DLL1, HES1*	-
regulation of epithelial cell proliferation	*LAMC1, PTPRK, EGFR*	*PTPRK, TSC2, FBXW7, FGFR2, MTSS1, SULF1, PTPRM, MMP12, LRP6, NRP1, SAV1, XDH, EGFR, TBX1*
positive regulation of epithelial cell proliferation	*LAMC1, EGFR*	*LAMC1, EGFR*
connective tissue development	*MATN1, SULF1, SPG20, LRP6*	*MATN1, SULF1, SPG20, LRP6,*

**Table S5.** (continued)		
**GO term**	**Up-regulated DEGs**	**Down-regulated DEGs**
epithelial cell-cell adhesion	-	*CDC42*
epidermis morphogenesis	-	*RUNX1, FGFR2, CDC42*
epidermal cell differentiation	-	*JAG1, CDC42, SAV1, CLIC4, RAC1*
epidermis development	-	*RUNX1, JAG1, FGFR2, CDC42, LRP4, SAV1, CLIC4, RAC1, EGFR*
epithelial cell development	-	*FGFR2, CDC42, CLIC4, RAC1, MYO1E*
epithelial cell migration	-	*MYH9, NRP1*
morphogenesis of an epithelial sheet	-	*JAG1, MMP12, LRP6*
epithelial-mesenchymal cell signaling	-	*CDC42*
epithelial cilium movement	-	*RFX3*
epithelial cell maturation	-	*RFX3*
glandular epithelial cell development	-	*RFX3*
mammary gland epithelial cell proliferation	-	*STAT6, ESR1, MED1*
hematopoietic stem cell differentiation	-	*XRCC5, ACE, TAL1, SRF, ERCC2*
regulation of epithelial cell differentiation	-	*XDH, SMO, DMBT1, PROM1, SOX2, APC, PAX2, VDR, NODAL, MED1, CAV1, RFX3, XDH, EZH2, KEAP1*
stem cell population maintenance	-	*SMC3, RIF1, SOX2, APC, PCM1, SKI, MED27, RTF1, SMC1A, NODAL, MED21, VPS72, GATA2, SRRT, LEO1, SRRT, FGFR3*
positive regulation of epithelial cell differentiation	-	*DMBT1, PROM1, SOX2, APC, PAX2, VDR, MED1, RFX3*
regulation of stem cell population maintenance	-	*SMO, CNOT1, PAX2, CNOT2, TAL1, NODAL, CNOT3*
neuronal stem cell population maintenance	-	*SOX2, PCM1, SRRT*
morphogenesis of a branching epithelium	-	*TCF21, LRP6, SALL1, SLIT2, PKD1, FGFR2, MET, FOXD3, LAMA1, SOX2, DLG1, WNT4, PAX2, VDR, ESR1, TBX20, IHH, GRB2, RBM15, MED1, MKS1, FOXA2, SRF*

Table S6. Gene symbol with full gene name

Gene symbol	Full gene name
ACE	Angiotensin-converting enzyme
APC	Adenomatous polyposis coli protein
AR	Androgen receptor
ASPM	Abnormal spindle-like microcephaly-associated protein
CASP8	Caspase-8 subunit p10
CAV1	Caveolin-1
CDC42	Cell division control protein 42 homolog
CLIC4	Chloride intracellular channel protein 4
CNOT1	CCR4-NOT transcription complex subunit 1
CNOT2	CCR4-NOT transcription complex subunit 2
CNOT3	CCR4-NOT transcription complex subunit 3
DLG1	Disks large homolog 1
DLL1	Delta-like protein 1
DMBT1	Deleted in malignant brain tumors 1 protein
DMD	Dystrophin
DOCK7	Dedicator of cytokinesis protein 7
EGFR	Epidermal growth factor receptor
EHF	ETS homologous factor
ENAH	Protein enabled homolog
EP300	Histone acetyltransferase p300
ERCC2	TFIIH basal transcription factor complex helicase XPD subunit
ESR1	Estrogen receptor
EZH2	Histone-lysine N-methyltransferase EZH2
FBXW7	F-box/WD repeat-containing protein 7
FGFR1	Fibroblast growth factor receptor 1
FGFR2	Fibroblast growth factor receptor 2
FGFR3	Fibroblast growth factor receptor 3
FOXA2	Hepatocyte nuclear factor 3-beta
FOXD3	Forkhead box protein D3
FZD1	Frizzled-1
GATA2	Endothelial transcription factor GATA-2
GRB2	Growth factor receptor-bound protein 2
GRHL2	Grainyhead-like protein 2 homolog
HES1	Transcription factor HES-1
IHH	Indian hedgehog protein
ILK	Integrin-linked protein kinase
IPMK	Inositol polyphosphate multikinase
JAG1	Protein jagged-1
KEAP1	Kelch-like ECH-associated protein 1
LAMA1	Laminin subunit alpha-1
LAMC1	Laminin subunit gamma-1

Table S6. (continued)

Gene symbol	Full gene name
LBX1	Transcription factor LBX1
LEO1	RNA polymerase-associated protein LEO1
LRP4	Low-density lipoprotein receptor-related protein 4
LRP6	Low-density lipoprotein receptor-related protein 6
MAF	Transcription factor Maf
MATN1	Cartilage matrix protein
MED1	Mediator of RNA polymerase II transcription subunit 1
MED21	Mediator of RNA polymerase II transcription subunit 21
MED27	Mediator of RNA polymerase II transcription subunit 27
MEF2A	Myocyte-specific enhancer factor 2A
MET	Hepatocyte growth factor receptor
MKS1	Meckel syndrome type 1 protein
MMP12	Macrophage metalloelastase
MSX2	Homeobox protein MSX-2
MTSS1	Metastasis suppressor protein 1
MYH9	Myosin-9
MYO1E	Unconventional myosin-Ie
NODAL	Nodal homolog
NRP1	Neuropilin-1/Protein kinase C-binding protein NELL1
PAK1	Serine/threonine-protein kinase PAK 1
PAX2	Paired box protein Pax-2
PCM1	Methyl-CpG-binding domain protein 1
PEX13	Peroxisomal membrane protein PEX13
PKD1	Polycystin-1
PRKDC	DNA-dependent protein kinase catalytic subunit
PROM1	Prominin-1
PTK7	Inactive tyrosine-protein kinase 7
PTPRK	Receptor-type tyrosine-protein phosphatase kappa
PTPRM	Receptor-type tyrosine-protein phosphatase mu
RAB10	Ras-related protein Rab-10
RAC1	Ras-related C3 botulinum toxin substrate 1
RBM15	Putative RNA-binding protein 15
RFX3	Transcription factor RFX3
RGMA	Repulsive guidance molecule A
RIF1	Insulin-like peptide INSL6/Telomere-associated protein RIF1
RSPO2	R-spondin-2
RTF1	RNA polymerase-associated protein RTF1 homolog
RUNX1	Runt-related transcription factor 1
SALL1	Sal-like protein 1
SAV1	Protein salvador homolog 1

Table S6. (continued)

Gene symbol	Full gene name
SFRP5	Secreted frizzled-related protein 5
SKI	Ski oncogene
SLIT2	Slit homolog 2 protein
SMC1A	Structural maintenance of chromosomes protein 1A
SMC3	Structural maintenance of chromosomes protein 3
SMO	Smoothened homolog
SOX2	Transcription factor SOX-2
SPG20	Spartin
SRF	Serum response factor
SRRT	Serrate RNA effector molecule homolog
STAT6	Signal transducer and activator of transcription 6
STRC	Stereocilin
SULF1	Extracellular sulfatase Sulf-1
TAL1	T-cell acute lymphocytic leukemia protein 1
TBX1	T-box transcription factor TBX1
TBX20	T-box transcription factor TBX20
TCF21	Transcription factor 21
TEAD1	Transcriptional enhancer factor TEF-1
TGM1	Protein-glutamine gamma-glutamyltransferase K
TSC2	Tuberin
VDR	Vitamin D3 receptor
VPS72	Vacuolar protein sorting-associated protein 72 homolog
WNT2B	Protein Wnt-2b
WNT4	Protein Wnt-4
XDH	Xanthine dehydrogenase/oxidase
XRCC5	X-ray repair cross-complementing protein 5

Additional file Figure:

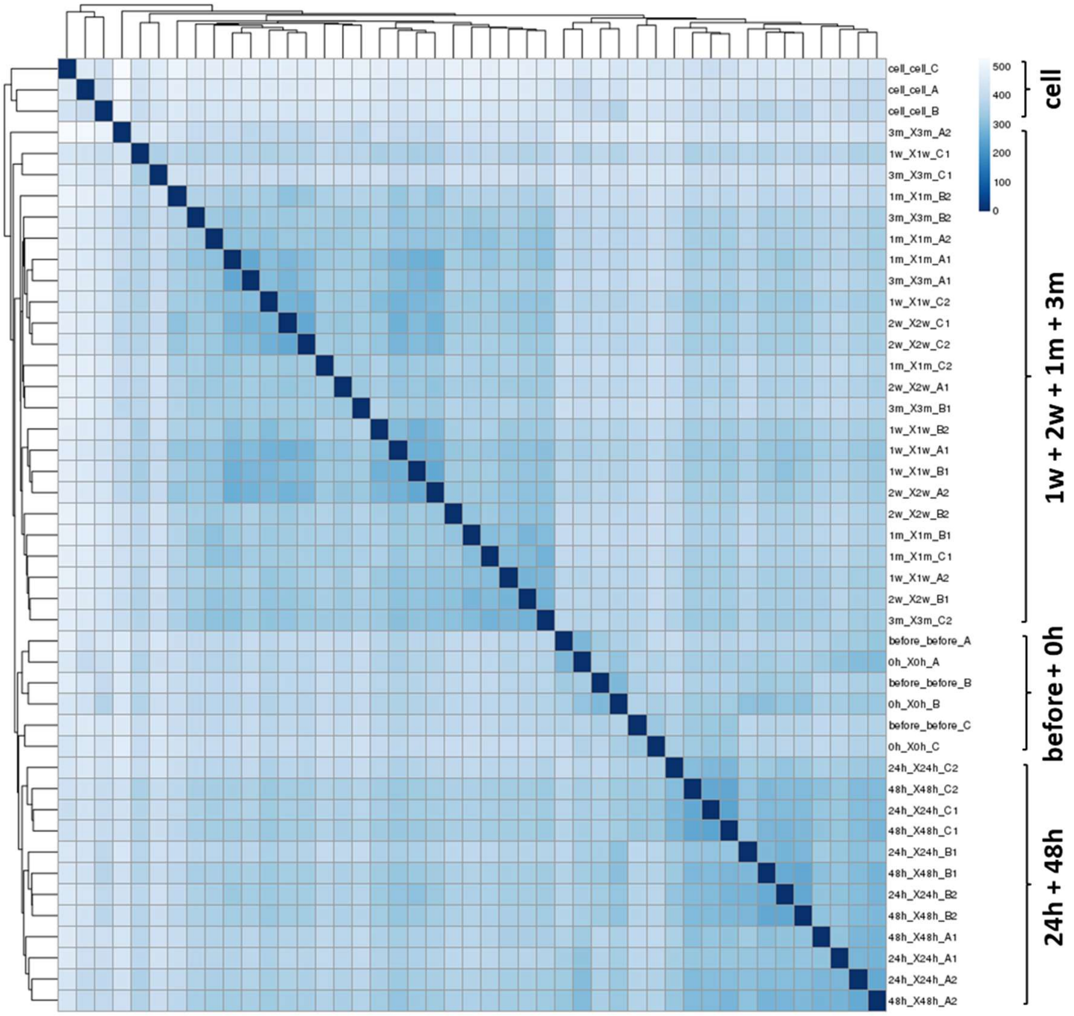

Figure S1. Heat map demonstrating whole gene expression profile at different stages of pearl grafting. Hierarchical clustering divided all the samples into four groups (cell, before + 0 h, 24 h + 48 h and 1 w + 2 w + 1 m + 3 m) as indicated on figure. Colour scale indicates the differences in expression.

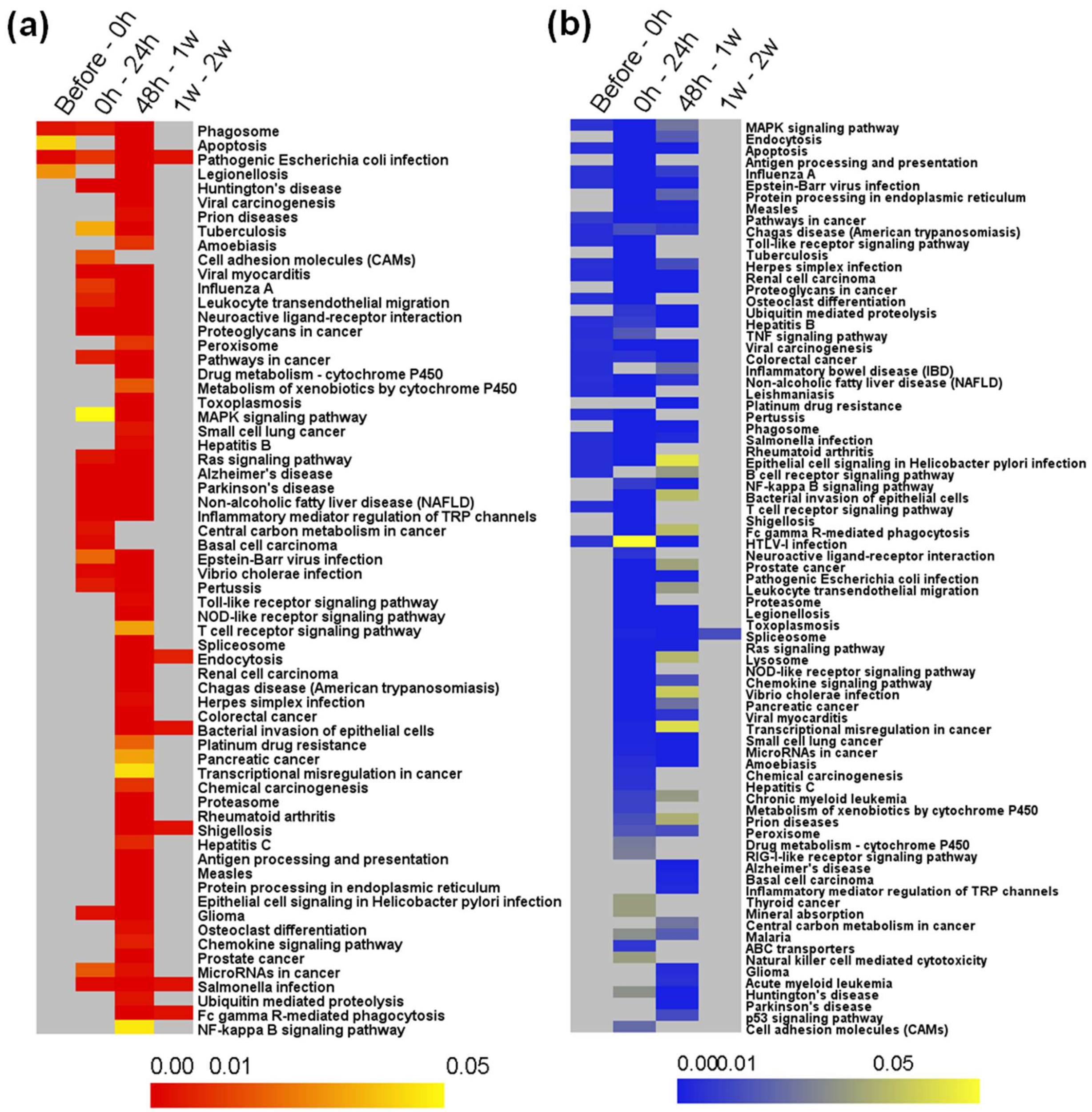

Figure S2. Heat maps of KEGG pathway enrichment analysis for immune related DEGs estimated by sleuth. (a) Up-regulated DEGs. (b) Down-regulated DEGs. Up- or down-regulated DEGs at each time point were submitted to KEGG pathway analysis using Kobas 3.0 web-based software. Columns and rows in the heat maps indicate treatments and enriched pathway terms, respectively. Sample names are displayed above the heat maps. Color scales indicate *P* values of enrichment tests and gray cells represent an empty value or a value > 0.05.

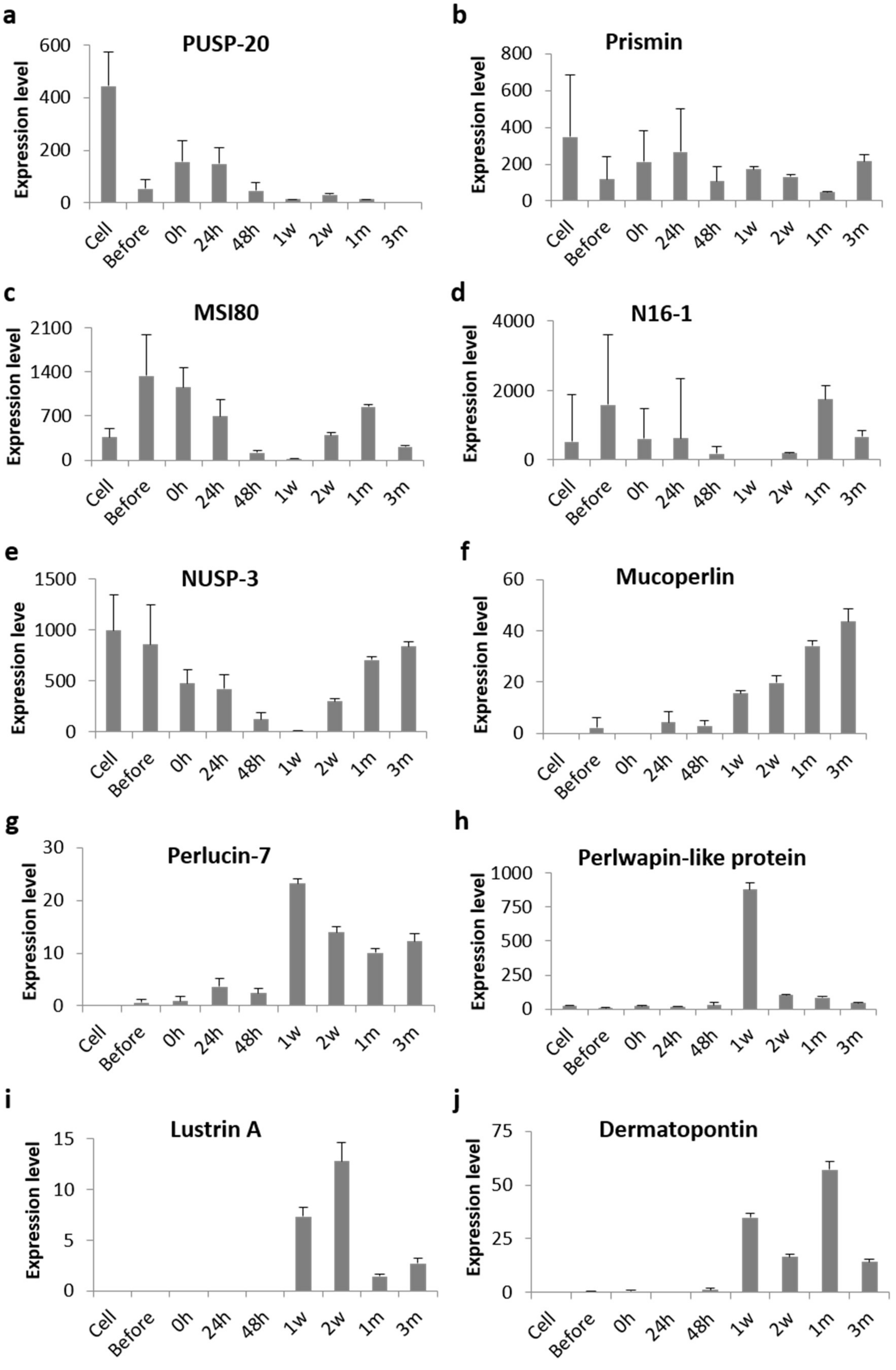

Figure S3. Expression patterns of SMPs involved in prismatic layer (a-b) and nacreous layer formation (c-j) at various time points of pearl sac and pearl development. Expression levels are indicated by adjusted TPM values (transcripts per kilobase million).

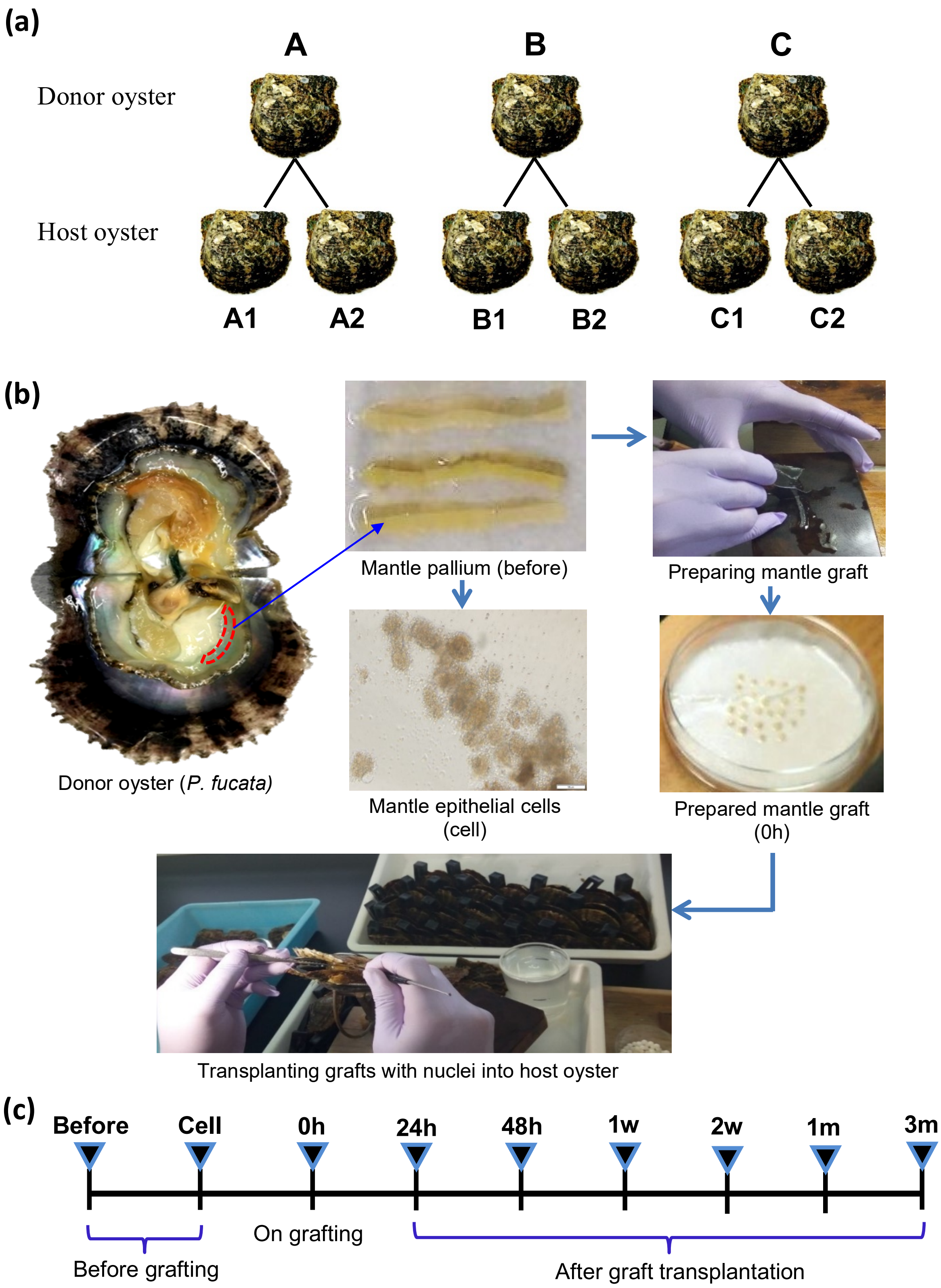

Figure S4. Experimental design for mantle grafting. (a) donor and host oysters used for grafting, (b) grafting process, and (c) sampling schedule. (b) Briefly, a pallial zone of mantle was excised from the pearl oysters and sterilized. The pallium strips were then used to prepare the grafts as well as to collect the epithelial cells, separately.

Additional references:

1. Kintsu H, Okumura T, Negishi L, Ifuku S, Kogure T, Sakuda S, et al. Crystal defects induced by chitin and chitinolytic enzymes in the prismatic layer of *Pinctada fucata*. Biochem Biophys Res Commun. 2017;489:89-95.
2. Wang Q , Yang C, Hao R, Zheng Z, Y Jiao, Du X , et al. Molecular characterization of CHST11 and its potential role in nacre formation in pearl oyster *Pinctada fucata martensii*. Electron J Biotechnol. 2017;28:113-119.
